# Revealing erythropoietin variant EV-3 as novel driving force and immunotherapeutic target in human glioblastoma

**DOI:** 10.1186/s13046-025-03620-3

**Published:** 2025-12-26

**Authors:** Stefania Elena Navone, Giovanni Marfia, Laura Guarnaccia, Massimiliano Rizzaro, Giorgio Fiore, Rolando Campanella, Chiara Gaudino, Daniele Santini, Giovanni Andrea Alotta, Monica Rosa Miozzo, Emanuela Barilla, Marco Locatelli, Laura Riboni

**Affiliations:** 1 Fondazione IRCCS Ca’ Granda Ospedale Maggiore Policlinico, Center for Aerospace Medicine and Advanced Therapies - CeMATA - Laboratory of Experimental Neurosurgery and Cell Therapy, Unit of Neurosurgery, Milan, Italy; 2 University of Milan, Department of Health Sciences, Milan, Italy; 3 University of Milan, Department of Pathophysiology and Transplantation, Milan, Italy; 4 Andremacon Srl, Milan, Italy; 5 Azienda Ospedaliero Universitaria Policlinico Umberto I, Department of Neuroradiology, Rome, Italy; 6 Sapienza University of Rome, Department of Medico-Surgical Sciences and Biotechnologies, Rome, Italy; 7 Azienda Ospedaliero Universitaria Policlinico Umberto I, Division of Oncology, Rome, Italy; 8 University of Milan, Medical Genetics Unit, Department of Health Sciences, ASST Santi Paolo e Carlo, Milan, Italy

**Keywords:** Erythropoietin, EV-3, Glioblastoma, Tumor microenvironment, Cancer immunotherapy, Cancer stem cells, Monoclonal antibodies, Hypoxia

## Abstract

**Background:**

Glioblastoma is the most aggressive primary brain tumor, and, despite intensive studies, remains one of the most fatal malignancy in adult humans. Among multiple onco-promoters produced by glioblastoma cells, erythropoietin was found. However, the presence/function of Epo alternatively spliced variants in cancer remains unexplored. Here, we investigated the expression and role of Epo-variants in glioblastoma, and the therapeutic potential of their targeting through a novel monoclonal antibody (mAb).

**Methods:**

Transcripts and protein levels of Epo-variants in a cohort of human brain tumors were evaluated by RT-PCR, ELISA, and immunohistochemistry. Monoclonal antibodies targeting Epo-Vs were prepared and functionally selected by assaying proliferation, migration, stemness, and angiogenesis in glioblastoma patient-derived cells. Antibody affinity for Epo/Epo-variant was determined by SPR. In vivo toxicity and therapeutic efficacy of the lead antibody were evaluated in GBM mouse models.

**Results:**

We found a significant overexpression of Epo-variant transcripts in tissues and cells from GBM patients. After functional selection of newly-produced antibodies, we identified AND-C4 as the lead one for its potent anti-tumoral properties, absence of anti-erythropoietic effects and of toxicity on human brain cells. AND-C4 exhibited high affinity for the Epo-variant EV-3. We demonstrated that EV-3 was efficiently produced and secreted by glioblastoma cells, particularly by stem cells. EV-3 exerted tumorigenic, angiogenic and immunomodulatory properties, and AND-C4 was effective in antagonizing all these actions. In vivo studies in rodent glioblastoma models revealed that AND-C4 selectively bound to tumor tissue and exhibited significant efficacy on tumor growth and animal survival.

**Conclusion:**

This study represents the first evidence on the presence, origin and pro-tumoral activity of EV-3 in human glioblastoma. Moreover, in vitro and in vivo results revealed AND-C4 as novel and promising anti-glioblastoma immunotherapeutic.

**Graphical Abstract:**

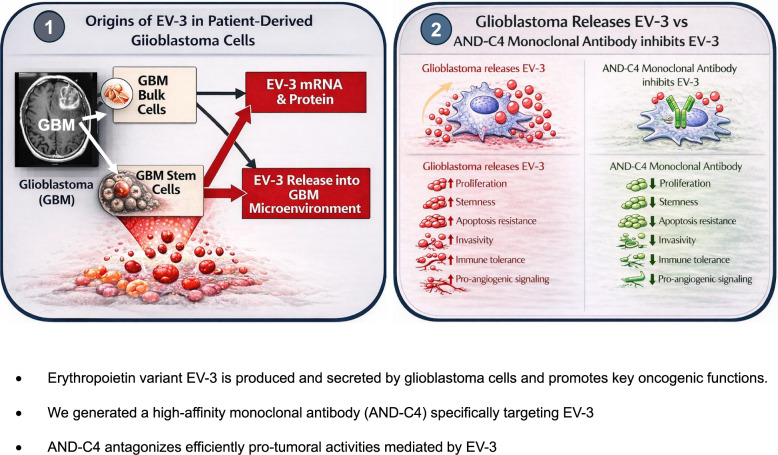

**Supplementary Information:**

The online version contains supplementary material available at 10.1186/s13046-025-03620-3.

## Background

Glioblastoma (GBM) is the most frequent and aggressive primary malignancy of the Central Nervous System (CNS) in adult humans [1, 2]. Of note, despite intensive research, significant advances in our knowledge, and multiple phase III trials, little has successfully translated into effective clinical treatments, leaving GBM as a lethal, orphan cancer. The evidence that the current standard of care is that proposed 20 years ago [3], and the median survival of GBM patients remains exceedingly poor (about 15 months), with an overall 5-year survival rate being one of the lowest (4–5%) among all cancer types [1, 4], underlines this therapeutic failure, and underscores a high unmet clinical need.

Different GBM features appear crucial in conferring a remarkable negative impact on patient prognosis, including: (i) extensive intra-tumoral heterogeneity, with the presence of a complex of genetically and functionally distinct cells [5, 6]; (ii) a specialized microenvironment, with multiple autocrine and paracrine signals generated by the interaction and cooperation not only of malignant cells, but also of brain and immune cells, fostering tumor growth, angiogenesis, immune evasion, and resistance to therapy [7–11]; and (iii) an extensive plasticity, where a small subpopulation of tumor cells, called GBM stem-like cells (GSCs), exhibits extraordinary properties of self-renewal, niche-shaping ability, hypoxia adaptation, and resistance to therapies. GSCs orchestrate the GBM niche by engaging a synergic and intensive relationship with the surrounding microenvironment, with hypoxia being a pivotal factor [8, 12–14]. These cells govern processes such as cell cycle regulation, stress response adaptation, and angiogenesis, all contributing to the malignant and recurrent nature of gliomas [15].

Among different molecules found in the GBM microenvironment erythropoietin (Epo) was recently identified [16]. Epo is an evolutionarily conserved hormone, widely recognized as the major promoter of erythropoiesis in the bone marrow [17, 18]. Multiple studies have shown that Epo functional significance goes beyond this, as it emerged to possess non-hematopoietic pleiotropic functions, including promotion of cell proliferation, prevention of programed cell death, modulation of metabolism and immunity, and tissue protection from stresses [19, 20]. The multiple effects of Epo appear to occur mainly through its binding to specific receptors. The major receptor for Epo (EpoR) is a homodimeric glycoprotein of the superfamily of cytokine receptors, abundant in bone marrow progenitors, and responsible for the erythropoietic effects [17]. Different functional variants of EpoR have been identified in the nervous system, including the colony-stimulating factor 2 receptor-β (CSF2RB), ephrin-type B receptor 4 (EphB4), and the cytokine receptor-like factor 3 (CRLF3) [21, 22]. However, it remains unclear the specific role(s) of these receptors in the different non-erythropoietic functions of Epo.

A poorly understood aspect of non-hematopoietic Epo refers to its heterogeneous nature, including the existence of different structural Epo variants (Epo-Vs). Indeed, the *EPO* gene codes not only for the classical Epo, but also for different proteins with qualitative/quantitative differences in their amino acid sequence, and generated from transcriptional variants produced by alternative splicing mechanisms. Alternative splicing is a nuclear process performed by the spliceosome (a ribonucleoprotein enzyme machinery made by multiple small nuclear ribonucleoproteins) that recognizes splice sites of pre-mRNA and produces, from a single gene, several mature mRNAs [23]. Defects in alternative splicing are frequently present in cancer, where they contribute to cancer progression and drug resistance [24]. Recent research has shown that the spliceosome is altered in GBM, with oncogenic splicing events promoting tumor development and aggressiveness [25–27].

Epo-Vs appear to exhibit a range of functional properties distinct from the erythropoietic action, possibly through binding to receptors different from the classical Epo-R [28]. Of relevance, Bonnas et al. described Epo-Vs as physiological components of human blood, with the major alternative splicing mechanism leading to the loss of exon 3, and translating into a glycoprotein, called EV-3, which is structurally and functionally different from the classical Epo [28].

Notably, experimental and clinical studies showed that the administration of pharmacological doses of recombinant human Epo (rhEpo), or its synthetic derivatives (erythroid stimulating agents, ESAs), for treating anemia in cancer patients showed that this treatment promotes tumor progression in many types of cancer, and is associated with worsened patient survival [29–32]. These series of evidence on Epo-/ESA-treated patients have been apparently corroborated by experimental investigations supporting that Epo may play a role in controlling cancer cell properties. The *EPO* gene was shown to exhibit increased expression in different solid tumors, and this appeared closely associated with the malignant features of cancers [33]. The expression of both Epo and/or its homodimeric receptor (Epo-R) has been recognized in a variety of human cancers, and rhEpo/ESA have been shown to foster progression, immune tolerance, angiogenesis, and apoptosis resistance in different tumors [32–34], encompassing glioblastoma (GBM) [35–41]. Different studies showed that GBM express both Epo and its receptors [37–41], particularly in hypoxic areas and invasive margins, and their expression is controlled by the hypoxia inducible factor (HIF) HIF1α [42]. However, conflicting data on both Epo, Epo-R levels and their role in cancer have been reported, and reported, and revealed that the use of nonspecific anti-Epo and anti-EpoR antibodies, as well as of improper methodologies and of excessive Epo doses impaired many of the reported findings in different tumors, including those in GBM [43–50].

A major lack of our knowledge on the oncogenic actions of Epo resides in the fact that the transcriptional variants of Epo have not been considered in the studies on human cancers, including those on GBM. This gap is of significant importance considering the possible oncogenic role of Epo/Epo-Vs, as well as the urgent need for new insights into GBM molecular characteristics for progress in its targeted therapy.

Starting from these premises, this study was undergone with the major aim to investigate the presence and role of Epo-Vs in GBM, as well as the potential effect of their targeting on GBM malignancy.

## Methods

### rhEPO and rhEV-3 protein production

cDNA of both *EPO* and hS3 with TwinStrep-tag at 3’/Cter and signal peptide for secretion at 5’/Nter were chemically synthesized with codon optimization for mammalian systems, and then sub-cloned in ProteoGenix’s proprietary optimized expression vector. The antigenic proteins were obtained from the purification of cell culture media, native protein extracts by HEK293 cells lysis in Phosphate-Buffered Saline (PBS), pH 7.5, and denatured protein extracts by solubilizing the cell pellet in 8 M urea. Purified proteins were submitted to SDS-PAGE in 12.5% polyacrylamide gels, and transferred to a nitrocellulose membrane for western blotting, according to the manufacture’s protocol (Bio-Rad, USA).

### Patients and tumor samples

Seventy patients with newly diagnosed brain tumors who underwent surgery for tumor excision at the Neurosurgery Unit of Fondazione IRCCS Ca’ Granda Ospedale Maggiore Policlinico from 2019 to 2023 were enrolled in this study. These patients included 10 cases of meningioma (MNG), 10 cases of grade 2 astrocytoma, called low-grade glioma (LGG), and 50 cases of GBM. The diagnosis was made by two independent pathologists and was updated according to the 2021 WHO classification of tumors of the CNS [1]. The Institutional Review Board approved the protocol (IRB#1670/2015), and all patients provided informed consent. Exclusion criteria were: (1) history or presence of other malignancies; (2) concomitant life-threatening disease. Demographic and clinical data of GBM patients were collected from patient records and are shown in Table 1. At the time of surgery, specimens of tumor samples were washed in Dulbecco-PBS, and dry frozen at − 80 °C for successive analyses.

**Table 1:** Demographic, clinical and molecular features of patients

Diagnosis	MNG (*n*, 10)	LGG (*n*, 10)	GBM (*n*, 50)
Sex			
Male, *n* (%)	2 (20)	9 (90)	33 (66)
Female, *n* (%)	8 (80)	1 (10)	17 (34)
Age, years			
median (IQR)	74 (74–75)	41 (36–59)	61 (51–70)
OS, months			
median (IQR)	n.a.	55 (36–84)	13 (7–25)
KPS, %			
median (IQR)	n.a.	100 (90–100)	80 (70–90)
Ki-67, %			
median (IQR)	5 (2–7)	5 (3–15)	30 (25–40)
MGMT, %			
median (IQR)	n.a.	18 (13–38)	14 (4–34)
IDH, n (%)			
wild-type	n.a.	0 (0)	50 (100)
mutated	n.a.	10 (100)	0 (0)

Data are shown as count and percentage) or as median and interquartile range (IQR). MNG: meningioma patients; LGG: low-grade glioma patients; GBM: glioblastoma patients; OS: overall survival; KPS: Karnofsky performance status; MGMT: O6-methylguanine-DNA methyltransferase; IDH: isocitrate dehydrogenase; n.a., not available.

### Cell cultures and peripheral blood mononuclear cells

Cell lines derived from human brain, including Neuronal Primary Cells (NPCs) (36057-01), and Neural Progenitor Stem Cells (36057-02), both from CelProgen (Torrance, CA, USA), and Astrocytes (ASTs) from Applied Biological Materials (Richmond, BC, Canada) were cultured following manufacturer’s instruction. U87MG-luc2 cells (PerkinElmer, Waltham, MA, USA), a cell line derived from a human GBM, and stably transfected with firefly luciferase gene (luc2), were cultured in Eagle’s Minimum Essential Medium (Thermo Fisher Scientific, Waltham, MA, USA) with 10% fetal bovine serum (FBS) (EuroClone, Milan, Italy), and 1% penicillin/streptomycin (Pen/Strep).

Primary GBM cells were obtained from ten GBM patient tissues and consisted in the followings: bulk of GBM cells (GBMCs), GSCs, and GBM-derived endothelial cells (GECs) (*n*=10 each). These cell cultures were established, cultured in different proper media, and characterized by immunphenotypic and molecular criteria as we previously described [51, 52].

All types of cell cultures were maintained in a humidified atmosphere of 5% CO_2_, 5% O_2_, at 37 °C. When specified, O_2_ levels were lowered to 1% to mimic a hypoxic condition.

Human CD34^+^ cells were isolated from human peripheral blood cells obtained from three healthy donors, using CD34 MicroBead Kit, and human CD34^+^ marker (Miltenyi Biotech, Germany), according to the manufacture’s protocol. CD34^+^ cells were plated in 6-well methylcellulose plates (Thermo Fisher Scientific, Waltham, MA, USA) (10^3^ cells/wells) with 2 ml of Methocult H4035 medium (Thermo Fisher Scientific, Waltham, MA, USA).

Peripheral blood mononuclear cells (PBMCs) were obtained from blood samples of three GBM patients, collected the morning before surgery in EDTA-tubes, and isolated by the Cytiva Ficoll-Paque™ PLUS protocol (EuroClone, Milan, Italy).

### In vivo studies on GBM mouse models

All experiments involving animals were performed in agreement with ethical standards, according to the Declaration of Helsinki, and were authorized by the Italian Ministry of Health.

In vivo studies were performed on 7-weeks-old female CD1 nude mice and included two GBM models: a patient-derived xenograft (PDX) model, and an orthotopic model with U87MG-luc2. All animals were maintained under specific pathogen free conditions, with constant T and humidity, and had ad libitum access to food and water.

For the PDX model, tumor specimens from a GBM patient were cut into 2–3 mm sized fragments and transplanted into the flanks of 34 anesthetized CD1 nude mice (Charles River Laboratories, Calco, Lecco, Italy) [53]. When tumor load reached ∼170 mm^3^, AND-C4 in PBS was intravenously injected at zero (placebo), 2, 5 and 10 mg/Kg, thrice a week for three weeks. The GBM orthotopic model was obtained by the intracranial injection of U87MG-luc cells (3 × 10^5^ in 5µl of PBS) in 16 nude mice [54]. AND-C4 (10 mg/kg per day) was intranasally administered (4 µl/nostril) to mice, after anesthetization with 3% isoflurane. The treatment schedule started 6 days after tumor implantation and was 3 times/day and 4 times/week for 3 weeks. All animals were observed daily for the entire experimental period.

Blood was collected after isoflurane anesthesia from the retro orbital plexus, or, before sacrifice, by submandibular venipuncture. Blood collection was in test tubes in either the absence or presence of EDTA. Whole blood from mice was freshly analyzed with HM5 Hemocytometer (Zoetis, Modena, Italy) and was used for both serum and plasma preparation. Serum was obtained by whole blood incubation (30 min, 20 °C), followed by centrifugation (1000 g, 10 min). Serum biochemical parameters related to kidney function (urea and creatinine), and liver function, albumin, aspartate aminotransferase (AST) and alanine aminotransferase (ALT) were measured through a biochemistry analyzer (ILab 650 Werfen, Barcelona, Spain). Plasma obtained from blood collected in EDTA-tubes was centrifuged (300xg, 10 min, 4 °C) and stored at -80 °C for AND-C4 quantification (see below).

In the PDX model, tumor size was measured with a Vernier caliper and evaluated by 7T Magnetic Resonance Imaging (MRI) (Biospec 70/30, Bruker MRI, Milan, Italy). Axial T2 weighted (T2w) and axial diffusion weighted (DWI) images were acquired on both controls and AND-C4 treated animals. Three experienced radiologists analyzed T2 sequences. The presence of hypointense fibrotic tissue inside the tumor was identified by drawing a free hand region of interest (ROI) on Horos visualization Software.

In animals with orthotopic GBM model, tumor growth was evaluated after i.p. injection of 10 µL/gr of D-Luciferin (PerkinElmer, Waltham, MA, USA), through BLI, using the IVIS Imaging System (PerkinElmer, Milan, Italy). BLI signals in the ROI were evaluated as Average Radiance, and expressed as mean photons/sec.

Mice body weight was recorded (2–3 times/week). Tolerability and potential toxicity of AND-C4 were evaluated by basic clinical observations, body weight loss, blood tests, and mortality. When clinical and behavioral abnormalities appeared, or at the end of the experiment, mice were sacrificed by CO_2_ inhalation. After sacrifice, brain, tumor, liver, and kidneys were collected. In mice with orthotopic GBM, brain tissue specimens were divided into three radial sections: anterior, middle and posterior (see Results). Tissue samples were snap frozen in dry ice (and stored at − 80 °C), and/or fixed in 10% formalin, and embedded in paraffin. For histological evaluation, tissue samples were stained with hematoxylin and eosin (H&E). To determine collagen deposition, Sirius Red Picrate (Bio-Optica, Milan, Italy) staining was performed following manufacturer’s instructions.

### AND-C4 biodistribution and Pharmacokinetic in mice

Frozen brain sections were suspended (0.1 g wet tissue/mL) in RIPA Lysis Buffer (Rockland Immunochemicals, Philadelphia, PA, USA) with Halt Protease Inhibitor Cocktail (Thermo Fisher Scientific, Waltham, MA, USA), and homogenized by the TissueLyser II (Qiagen, Hilden, Germany), at 30 Hz for 3 min. The homogenate was then clarified by centrifugation (20.000xg for 20 min, at 4 °C). The resulting brain extracts were gently transferred to clean tubes and subjected to AND-C4 quantification by enzyme-linked immunosorbent assay (ELISA) (see below).

### Immunohistochemistry

Immunohistochemical staining on mouse brain samples was carried out on formalin-fixed tissues using an anti-Ki-67 Ab (Clone Mib-1, Agilent Dako, Santa Monica, CA, USA) (1:100), as cell proliferation marker, and an anti-cleaved Caspase 3 mAb (Agilent Dako, Santa Monica, CA, USA) (1:500) as apoptosis marker. Images were captured by Nikon E800M microscope and DXM1200C digital camera with Image Pro Plus software (Media Cybernetics, Rockville, MD, USA). For Immunohistochemistry (IHC) quantification, 10^3^ cells from at least five separate tissue sections were counted, using ImageJ software. Caspase- 3 staining was calculated by counting positive cells in five high power fields (40x) in 5–8 randomly selected areas from each tumor. The number of positive, brown-stained cells over the total cell number was evaluated and used to determine the percent of staining. Immunohistochemical staining on human brain and tumor samples was performed on human paraffin-embedded tissue arrays (TissueArray.Com LLC, Derwood, MD, USA). Tissue samples included triplicate cores/case, each core measuring one mm in diameter, and 5 μm in section thickness. Sample origins were: normal brain tissue (*n* = 9 cases), LGG (*n* = 22 cases), and GBM (*n* = 23 cases). After paraffin removal and rehydration, overnight incubation with AND-C4 (5 µg/mL) at 4 °C followed. AND-C4 binding was revealed using the Polymer Detection kit (Cat# GAMHRP, Microtech S.r.l., Milan, Italy), and quantification of its expression was obtained by digital scanning with 3D Histech (Budapest, Hungary).

Spatial immunofluorescence was performed on paraffin-embedded tissue slices from GBM tissue samples. To expose target proteins, heat-induced epitope retrieval was performed by using Na-citrate buffer antigen retrieval solution (pH 6, 30 min at 95 °C), followed by incubation with blocking solution (2% goat serum in PBS), and then probe with AND-C4 antibody (as above) and HIF1α antibody (Invitrogen, Waltham, MA, USA, 1:1000) overnight at 4 °C. Detection was performed using goat anti-mouse IgG Alexa Fluor plus 488 (1:200) and donkey anti rabbit cy3 (H + L) highly cross-adsorbed (1:200) secondary antibodies (60 min, 20 °C). Nuclei were stained with DAPI, and the sections were mounted using PBS/Glycerol. Microscopic images were visualized by Olympus Upright BX51 Full Manual.

### Quantitative real-time PCR analyses

Tissue specimens (~ 15 mg wet weight) and different cells (2 × 10^6^) were homogenized in 500 µL of TRI-Reagent Solution and submitted to total RNA extraction and quantification with NanoDrop 1000 Spectrophotometer (Thermo Fisher, Waltham, MA, USA). Reverse transcriptase reaction was performed by loading one µg of RNA, and using TranScriba Kit (A&A Biotechnology, Gdansk, Poland). Quantitative real-time PCR (qRT-PCR) was performed according to StepOnePlus™ (Thermo Fisher Scientific, Waltham, MA, USA). Primer sequences used to assess mRNA expression are reported in Table 2. The housekeeping gene ß-Actin (*ACTB*) was used as reference.

**Table 2:** Sequences of forward and reverse primers used for qRT-PCR

Transcript	Forward Sequence	Reverse Sequence	Tm (°C)	Amplicon length (bp)	Predicted Target
*ACTB*	ACTCCTGCTTGCTCATCCAC	CCACCATGTACCCTGGCATT	60	177	NM_001101.5
*EPO*	CCCAGAGCAGGAAGCATTCA	CTGTCCCTGATGGTAGGTGC	61	153	NM_000799.4
hs3	CCGAGAATATCACGGTCGGG	CGGCTTTATCCACATGCAGC	61	144	ntt
hs4	GAAGAGGATGGAGCCGTGG	ATCTGGAGGGGAGATGGCTT	60	124	ntt
h1-4	AGCAGGCCCTGTTGGTC	ATCTGGAGGGGAGATGGCTT	58	140	ntt
h1-5	CGAGAATATCACGACGGGCT	GTTGACCAACAGGGCATAGA	59	97	ntt

*Tm*, temperature of melting; *BP*, base pairs; *ntt*, no target template

### Monoclonal antibody production and functional selection

Anti-Epo monoclonal antibodies (mAbs) were generated by hybridoma technique (ProteoGenix, France). In detail, BALB/c mice (*n*=5) received subcutaneous injections of rhEpo (6 times, once every 2 weeks), supplemented with Freund’s adjuvant. After eleven weeks, spleen cells with the highest Ab titer, were collected and fused with Sp2/0 myeloma cells, creating hybridoma-producing mAbs. Hybridoma cells were cultured in RPMI 1640 with 10% FBS, 1% glutamine, and 1% Pen/Strep.

Twenty-two clones were identified through repetitive sub cloning by the limiting dilution technique and screening via ELISA assay, and four mAbs were produced from the selected clones. After protein A/G purification, mAbs were collected in PBS, and concentration was determined by spectrophotometry at 280 nm. These mAbs were then submitted to functional selection by assessing their effect first on erythropoiesis, and then on proliferation and apoptosis of brain and tumor cells. To this end, we performed the following assays:

1. colony forming unit (CFU). Human CD34 + cells were treated with rhEPO (10 IU/mL) in the absence or presence of different mAbs (2–20 µg/mL) for 2 weeks. Thereafter, colony-forming units were counted by ImageJ.
2. cell proliferation and apoptosis. In order to simultaneously screen the sensitivity of different cells (from human brain and GBM) to anti-cancer drugs, the Gliosphera platform assay was specifically developed. To this purpose, GBMCs, NPCs, NPSCs, or ASTs were seeded (5 × 10^3^/well) in a 96-well Gliosphera plate and treated with mAbs at different concentrations (2–20 µg/mL) for 96 h. The medium was then removed, and cell proliferation and apoptosis were assessed by 3-[4,5-dimethylthiazol-2-yl]-2,5 diphenyl tetrazolium bromide (MTT) assay (Thermo Fisher Scientific, Waltham, MA, USA), and by RealTime-Glo™ Annexin V Apoptosis Assay (Promega, Madison, WI, USA), respectively.

After these functional assays, one mAb called AND-C4 was selected. The purity of the AND-C4 mAb was determined by SDS-PAGE, and the SEC-HPLC method (Agilent, Santa Clara, CA, USA).

### Surface plasmon resonance

Surface Plasmon Resonance (SPR) was used for two purposes:

- Measurement of AND-C4 kinetic and affinity. The experimental approach involved the analysis of the direct interaction between immobilized AND-C4, or the anti-Epo antibody 16 F (16F1H11, StemCell Technologies, Vancouver, Canada), and flowing rhEPO or rhEV-3. The mAb was immobilized through an amine-coupling process onto the surface of a biosensor chip (SPP CMD50L, XanTec Bioanalytics). For this, the chip surface was activated by flowing a solution made by 50 mM hydroxysuccinimide, and 400 mM 1-ethyl-3-(3-dimethylaminopropyl-carbodiimide. Then, Ab (30 µg/mL in Na-acetate buffer, pH 5.0) was flowed, and the remaining activated C-groups were deactivated by flowing 1 M ethanolamine, pH 8.0. All flowing steps were at 30 µL/min, for 5 min. Binding studies were through ProteOn XPR36 Protein Interaction Array system (Bio-Rad Laboratories, Segrate, Milan, Italy). After chip rotation, the analytes (3-300 nM), dissolved in running buffer (PBS with 0.005% Tween 20, pH 7.4), were flowed (as above) over immobilized ligands. Dissociation was monitored for the next 25 min. The SPR signals on the sensorgrams were expressed as Resonance Units (RU) and corrected by subtracting the non-specific response in the reference channel. The binding constants were obtained by fitting to entire sensorgrams (association and dissociation phases).
- Measurement of AND-C4 pharmacokinetic. The AND-C4 mAb concentration was determined in plasma and tissues (tumor, liver and kidneys) from mAb-treated mice (see above). To analyze the AND-C4 dependent SPR signals, tissues were homogenized in 2 mL/g of TBST buffer (75 mM Tris-Cl, pH 7,0, 450 mM NaCl, 1 mM EDTA, 1 mg/ml of Non-Specific Binding reducer (Cytiva, Marlborough, MA, USA), and 0.005% Tween 20) with a Precellys Lysing Kit (Thermo Fisher Scientific, Waltham, MA, USA). Samples were then ultracentrifuged (110.000xg for 1 h), and the supernatant was diluted (1:2) with the TBST buffer. rhEV-3 and bovine serum albumin (BSA) (used as reference) were diluted at 400 µg/mL and 30 µg/mL, respectively, in acetate buffer, pH 4.5, and immobilized using amine-coupling chemistry on parallel channels of a CMD700M (XanTec GmbH) sensor chip. The solutions flowed (5 µL/min for 10 min at 25 °C) over the activated chip surface, with TBST buffer as running buffer. The remaining activated C-groups were blocked as above. The analyte solutions (e.g. control plasma containing spiked AND-C4 mAb, or plasma samples from treated mice) were injected to simultaneously flow on either the reference surface or immobilized rhEV-3. Dissociation was measured in the following times (11–30 min). The sensorgrams (time course of the SPR signal in RU) were normalized to a baseline value of zero. The signals observed in the surfaces immobilizing rhEV-3 were corrected by subtracting the nonspecific response observed in the reference surfaces.

### ELISA assays for AND-C4 binding affinity and quantification of Epo, EV-3 and AND-C4

Epo and EV-3 quantification was performed on tumor tissues, tumor-derived cells and their conditioned medium. Initially, tumor samples were suspended (∼15 mg of wet weight/500 µL) in RIPA Lysis Buffer with Halt Protease Inhibitor Cocktail, and homogenized (Tissue Ruptur II, Qiagen). GBMCs and GSCs (2.5 × 10^5^ cells/2 mL, cultured in 6-well plates for 72 h) were lysed as above, and their conditioned medium was analyzed. Both solubilized tissue samples and lysed cells were centrifuged (8.000xg, 15 min at 4 °C), and supernatants were then used. EPO levels were determined using the Human Erythropoietin/EPO Quantikine ELISA Kit (R&D Systems), and EV-3 ones using a newly customized sandwich ELISA assay, as follows.

In the sandwich ELISA, 96-well clear flat bottom polystyrene high bind microplates (Thermo Fisher Scientific, Waltham, MA, USA) were coated with AND-C4 (1 µg/mL of 0.58 M Na_2_CO_3_/NaHCO_3_ buffer, pH 9.5), and this was followed by overnight incubation at 20 °C. After blocking with 3% BSA in PBS with 0.1% Tween 20 (PBST) for 1 h at 20 °C, either standard rhEV-3 (0.02-2 ng/mL), or tissue, or cells, or conditioned medium was added, and samples were incubated at (2 h, 20 °C). After 3 washings with PBST, a 2^ary^ anti-EV-3 IgG-HRP was added for 1 h at 20 °C. Finally, 3,3,5,5-tetramethylbenzidine (FineTest Biotech Inc., Boulder, CO, USA) was added (20 min at 20 °C in the dark), and the reaction was stopped with 50 µL/well of Stop Solution (FineTest Biotech Inc., Boulder, CO, USA). Absorbance was read at 450 nm with the Synergy H1 Microplate Reader (BioTek, Winooski, VT, USA). The sample concentration of EV-3 was calculated from calibration curves obtained by EV-3 spiking into each specific matrix (tissue/cell/medium).

AND-C4 quantification in mouse brain was performed by using 96 Clear Flat Bottom wells, coated with rhEV-3 (5 µg/ml, overnight at 4 °C) in 50 mM Na_2_CO_3_/NaHCO_3_ buffer, pH 9.5. After blocking with 3% BSA, brain extracts (diluted 1:5 w/v in PBS) were incubated at 37 °C for 1 h. In parallel, a calibration curve was run using AND-C4 (0.78–100 ng/mL), dissolved in matrix (brain homogenate from naïve mouse). An anti-mouse IgG-HRP, diluted 1:500 in PBS, was then incubated at 37 °C for 1 h. The following steps were performed as here above.

### Effects of rhEV-3 and/or AND-C4 on different cells of the GBM niche

The functional effects of EV-3 and AND-C4 were evaluated on the followings:

(1) Cell proliferation. GBMCs and GSCs were plated (5 × 10^5^ cells/mL) and incubated for 3 days. Thereafter, cells were treated with rhEV-3 (100 ng/mL), or AND-C4 (10 µg/mL), or their combinations for 96 h, and thereafter MTT assay was performed.

(2) Spheroid formation. GSCs were plated (1–5 × 10^5^ cells/mL) on Matrigel-coated dishes Corning^®^ (Thermo Fisher Scientific, Waltham, MA, USA), and incubated for 7–10 days. Then, GSCs were stained with calcein AM (Miltenyi Biotech, Germany) (1 mg/mL, 30 min at 37 °C). Then treatment with rhEpo (10 IU/mL), or rhEV-3 (100 ng/mL), or AND-C4 (10 µg/mL), or their combinations for 96 h followed. At the end, GSCs were analyzed by microscope (Leica wide field microscope, DMI6000B) and images were acquired in five random fields. Spheroid formation was quantified by measuring the mean diameter of individual spheroids.

(3) Cell migration. GBMCs and GECs (10^4^ cells) were seeded in each side of Ibidi culture-inserts (Ibidi GmbH, Grȁfelfing, Germany) and cultured until ∼95% confluence was reached. Inserts were then removed, and cells were incubated with rhEV-3 (100 ng/mL), AND-C4 (10 µg/mL), or their combination. After 24 h, cells were stained with calcein (1 mg/mL) for 30 min at 37 °C. Microscopic images of cells were acquired (inverted Leica DMI6000B wide field microscope), and cells migrated into the gap were counted through ImageJ.

(4) Angiogenesis. The angiogenic potential of GECs was evaluated by a tube-like structure formation assay, using µ-plate Angiogenesis 96-wells (Ibidi GmbH, Grȁfelfing, Germany), coated with 12.5 mg/mL matrigel, 10 µL/well on ice. After gentle agitation to ensure complete coating and matrigel solidification, plates were incubated at 37 °C for 30 min. Then, GECs (1 × 10^4^/well) were seeded and added with rhEV-3 (100 ng/mL), and/or AND-C4 (10 µg/mL), and incubated (48 h, at 37 °C). Cord formation was monitored with an inverted Eclipse Ti-E microscope (Nikon Instruments, Florence, Italy), equipped with a high-resolution cSMOS camera (Andor Zyla, Andor Technology, Belfast, UK) and NIS_Elements 4.51 software. After 48 h of incubation, five random images/well were acquired and analyzed with the Angiogenesis Analyzer plugin in ImageJ.

(5) Chemotaxis and invasion of PBMCs. Transwell chemotaxis chamber assays (Thermo Fisher Scientific, Waltham, MA, USA) were performed. PBMCs (10^4^ cells/100µL of culture medium) were added to the top compartment of the chamber and 400 µL of culture medium were added to the lower chamber in the absence or presence of rhEV-3 (100 ng/mL) and AND-C4 (10 µg/mL), alone or in combination. Following 48 h incubation, PBMCs migrated to the lower compartment were stained with calcein (1 mg/mL), and images were acquired with an invert Eclipse Ti-E microscope (Nikon Instruments, Florence, Italy) equipped with an Andor Zyla 5.5 sCMOS camera (Andor Technology, Belfast, UK). The number of migrated PBMCs in the whole well was obtained through the Analyze Particle plug-in of ImageJ.

### Statistical analysis

All statistical analyses were performed using SPSS version 29 (IBM, New York, NY, USA). The software was used to calculate individual mean, group mean, and standard deviation (SD). The normality of data distribution was assessed using the Kolmogorov–Smirnov test, and when data followed a normal distribution, differences between groups were evaluated using Student’s t-test or one-way analysis of variance (ANOVA). For data not conforming to a Gaussian distribution, the non-parametric tests Kruskal–Wallis and post hoc pairwise Mann–Whitney U two‐sided tests were used. Animal survival was evaluated along with time through Kaplan-Meier estimates, and statistical significance was analyzed using the Log-rank test (Mantel-Cox). Differences among groups were considered statistically significant for *P* < 0.05.

## Results

### *EPO *and Epo-V transcripts are over-expressed in GBM tissues and their derived cells

**Fig. 1:**
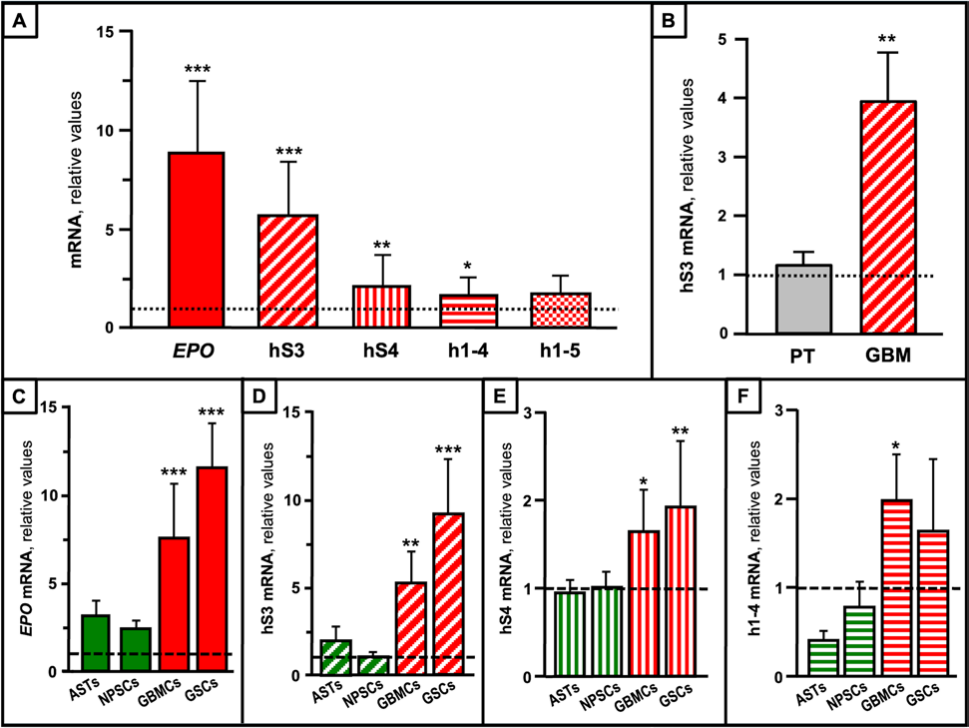
Expression of *EPO* and EPO-V transcripts in human GBM tissues and cells. **A **Transcript expression of *EPO* and its variants in tumor specimens from GBM patients (*n* = 50) relative to human brain (dotted line) (*n* = 3). Data are reported as mean ± SD. *, *P* < 0.05, **, *P* < 0.01, ***, *P* < 0.001 vs. human brain. **B **Transcript levels of hS3 in tumor specimens from GBM patients (*n*= 5), and cross-matched peritumoral tissue (PT) (*n* = 5). Data are reported as relative fold change to human brain (dotted line). **, *P* < 0.01 vs. PT. **C-F **Transcript expression of *EPO* (**C**), hS3 (**D**), hS4 (**E**), and h1-4 (**F**) in NPSCs (*n* = 3), ASTs (*n* = 3), GBMCs (*n* = 10), and GSCs (*n* = 10). Data are reported as relative to NPCs (dashed line) and as mean ± SD. *, *P* < 0.05, **, *P* < 0.01, ***, *P* < 0.001 vs. NPCs. All samples were quantified in triplicate

To investigate EPO-Vs in GBM tissues, and derived cells, we performed qRT-PCR. The results revealed a significant overexpression of the *EPO* mRNA, and of its transcriptional variants, including hS3, hS4, and h1-4 in GBM tissues, when compared to human adult brain (Fig. 1A). Notably, in GBM tissues, the hS3 transcript, which is translated into the EV-3 protein, was the most abundant mRNA, and was significantly overexpressed when compared to both human brain (Fig. 1A), and matched peritumoral tissues (Fig. 1B). Then transcripts of *EPO* and its variants were then investigated in cells from human brain and from patient-derived GBM tissues. The results demonstrated that mRNA levels of *EPO*, hS3, hS4, and h1-4 were rather similar in human brain cell lines (NPSCs, NPCs, and ASTs), but significantly higher in GBMCs and GSCs than in these brain cells (Fig. 1C-F).

### Functional selection of the lead anti-Epo/Epo-V mAb

**Fig. 2:**
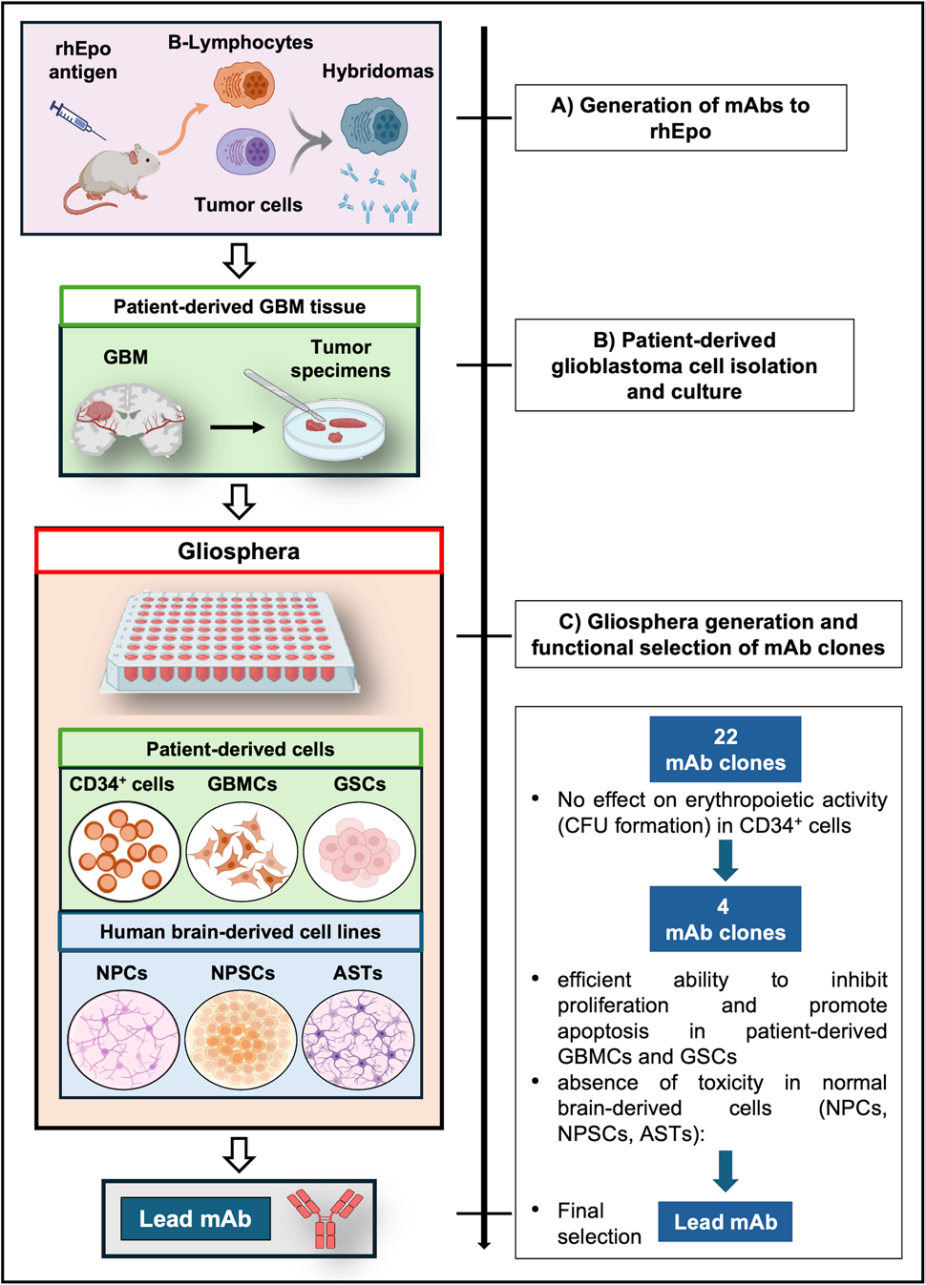
Flowchart of mAbs production and functional selection through the Gliosphera.** A **Monoclonal antibodies (mAbs) (*n* = 22 clones) were generated by hybridoma technique. **B **Patient-derived glioblastoma tissues were used to prepare cultures of glioblastoma cells (GBMCs), and glioblastoma stem-like cells (GSCs). (**C**) Patient specific platforms (Gliosphera) were prepared by seeding patient-derived blood and tumor cells (CD34^+^ cells from peripheral blood, GBMCs and GSCs), and normal cells derived from human brain (NPCs, NPSCs and ASTs). Functional selection followed by administering to cells the different mAbs, to finally obtain the lead mAb

In light of the marked upregulation of different *EPO-V* transcripts, we next sought to develop a mAb able to selectively inhibit the oncogenic properties of Epo/Epo-Vs, while preserving the erythropoietic activity. After hybridoma technique, we obtained 22 mAb clones and we then proceeded with a two-step functional selection, which led us to gradually obtain the most efficient, lead mAb. As shown in the flowchart of Fig. 2, step (1) of selection was performed to exclude anti-Epo mAbs able to inhibit rhEpo-induced CFU formation; step (2) was performed to simultaneously screen the sensitivity of different cells to AND-mAbs through a platform, called Gliosphera, which we specifically developed. This platform incorporated GBMCs and GSCs, as well as cell lines from human brain, i.e. NPCs, NPSCs and ASTs.

**Fig. 3:**
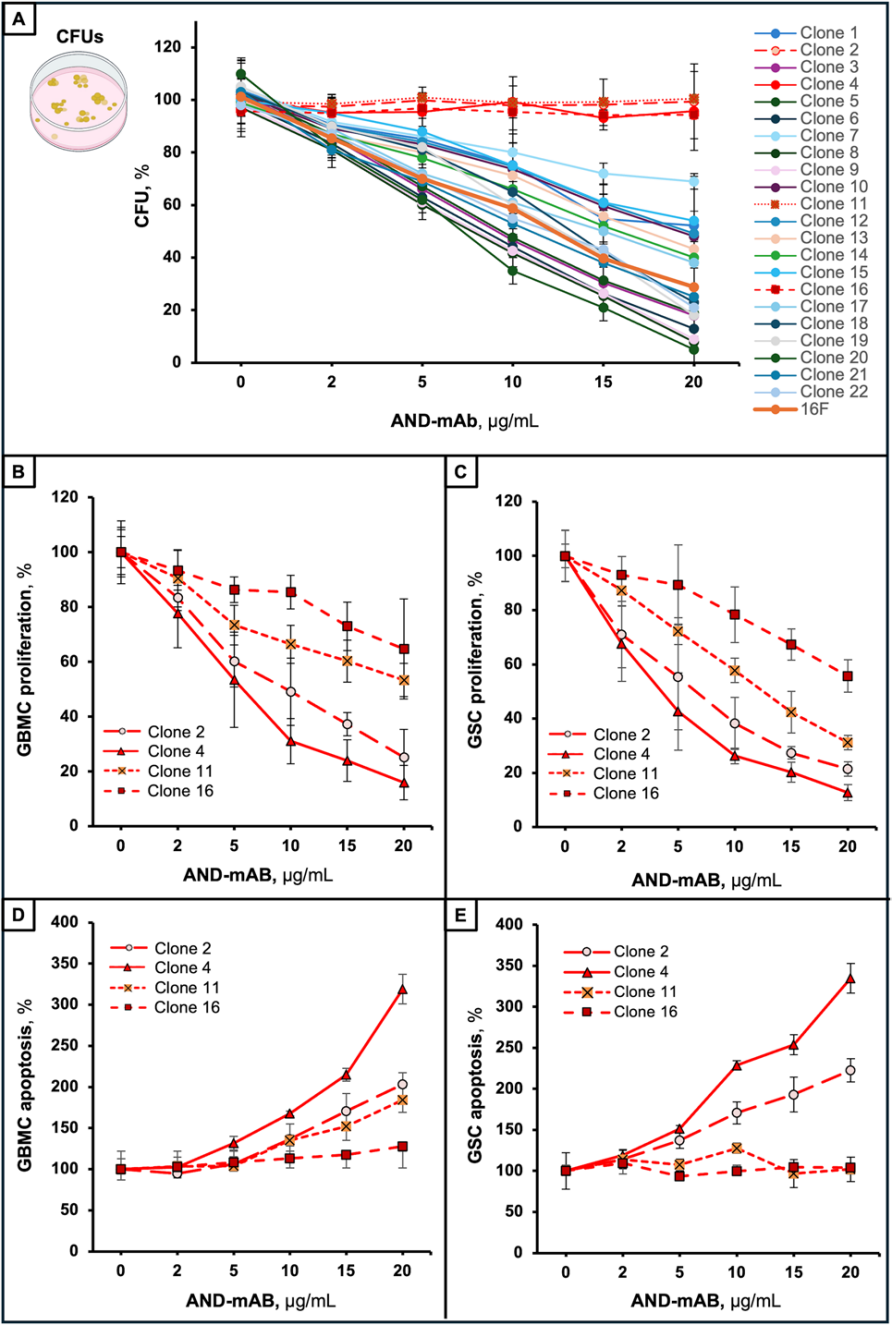
Functional selection of anti-Epo mAb clones. **A **Dose-response effect of the initial AND-mAb clones (*n* = 22) on CFU formation by CD34^+^ blood cells in the presence of rhEpo (10 IU/mL). The 16 F anti-EPO Ab was used as positive, inhibitory control (dark orange line). Data are reported as percent of control cells and are the mean of triplicate assays. **B** to **E **Dose-response effect of the AND-mAb clones (*n* = 4, selected in A) on proliferation **(B** and **C**) and apoptosis **(D** and **E)** of GBMCs (*n* = 3) (**B**,** D)** and of GSCs (*n* = 3) (**C**,** E**). Data are the mean ± SD of triplicate assays

The first step of selection allowed us to select 4 out of 22 AND-mAbs deprived of anti-erythropoietic action in the dose range of 2–20 mg/ml (Fig. 3A). In this selection 16 F, a commercially available anti-Epo Ab known to inhibit Epo-mediated erythropoiesis [53], was used as control. As expected, it exerted a dose-dependent inhibition of colony formation units (orange line in Fig. 3A). In the second step of selection, we used the Gliosphera platform to evaluate AND-mAbs potential and efficiency in counter-acting tumoral properties of three patient-derived GBM cells, including proliferation of GBMCs and apoptosis of GSCs. The parallel absence of negative effects/toxicity on the three types of human brain cells was the further leading criterion for lead mAb selection. Through the Gliosphera assay, among the four AND mAbs, the clone 4, thereafter called AND-C4, was finally selected as the most efficient anti-tumor mAb able to inhibit proliferation (Fig. 3B and C), and to promote apoptosis (Fig. 3D and E) of both GBMCs (Fig. 3B and D) and GSCs (Fig. 3C and E),

### Functional validation and quality control of the AND-C4 mAb

**Fig. 4:**
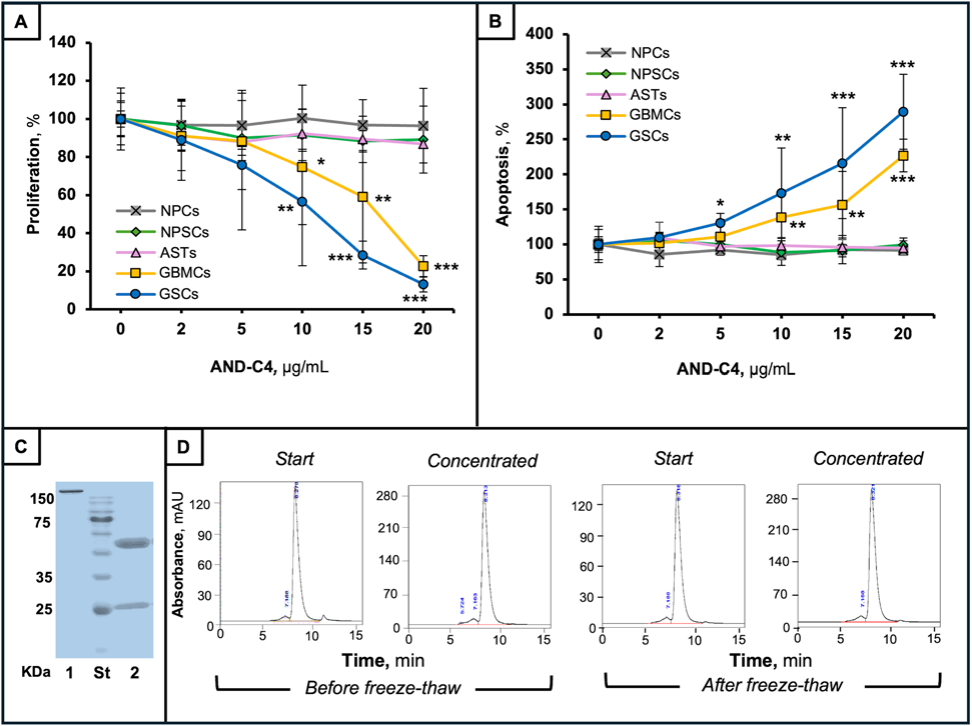
Functional validation and quality control of AND-C4 mAb. **A**,** B **Effect of different doses of AND-C4 on the proliferation **A** and apoptosis **B** of human brain-derived cells and GBM-derived cells (*n* = 10). Data are the mean ± SD of triplicate assays. *, *P* < 0.05; **, *P* < 0.01; ***, *P* < 0.001 vs. untreated cross-matched cells. **C **SDS-PAGE of AND-C4 prior (lane 1) and after (lane 2) reduction. St, standard MW. Samples were visualized by Coomassie blue staining. **D **SEC-HPLC profile of AND-C4 prior, and after concentration (left panels), as well as prior, and after freeze-thaw (right panels)

We then proceeded into a detailed functional validation of AND-C4 by assessing its effects on both proliferation and apoptosis in GBMCs and GSCs obtained from tissues of ten GBM patients. The results of this screening demonstrated that AND-C4 significantly inhibit cell growth and induced apoptosis in a dose-dependent fashion, in both cell types from all patients (Fig. 4A and B). To the opposite, at all effective doses, AND-C4 was unable to influence these processes in all the three types of human brain cells (Fig. 4A and B). We then assessed quality control of AND-C4. SDS-PAGE profiles showed a high purity (> 95%) of AND-C4 both prior and after reduction (Fig. 4C). SEC-HPLC analyses confirmed this purity, and showed that the purified mAb did not exhibit detectable degradation or aggregation after concentrations tests and/or freeze-thaw (Fig. 4D).

### AND-C4 binds EV-3 with high affinity

**Fig. 5:**
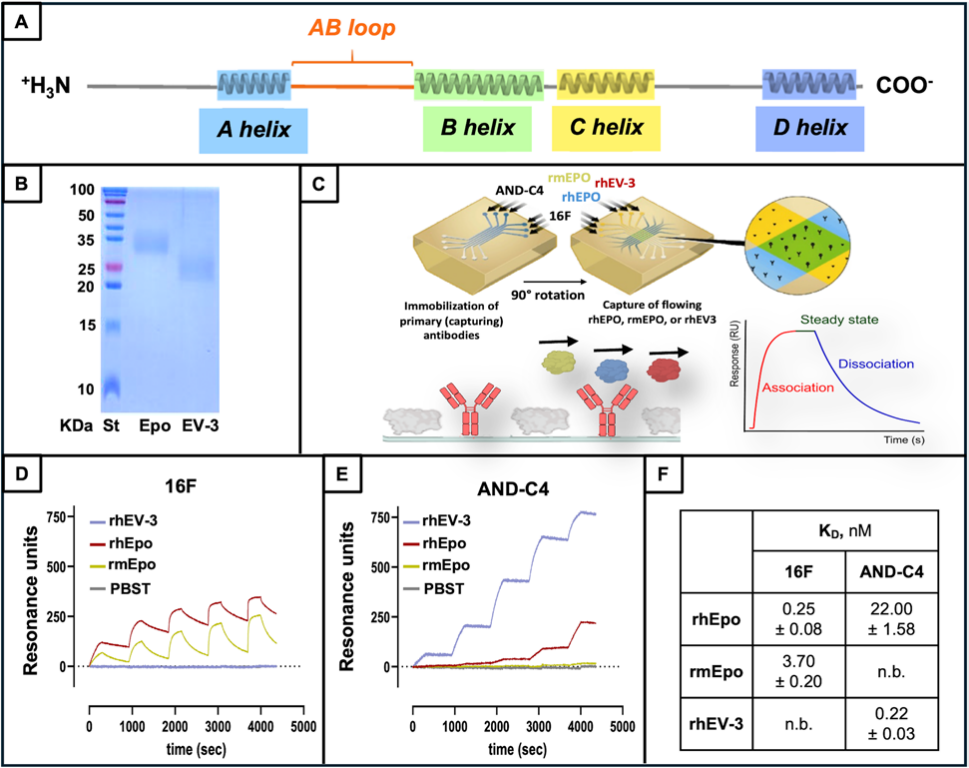
Structural and binding features of AND-C4 and EV-3. **A **Schematic representation of the Epo protein structure, with the AB loop lacking in EV-3 highlighted in orange. **B **SDS-PAGE of rhEpo and rhEV-3, visualized by Coomassie blue. St, standard MW. **(C)** Schematic representation of SPR workflow used to study the interaction between tested mAbs and their predicted ligands. **D** and **E **SPR sensorgrams obtained by flowing rhEV-3, rhEpo rmEpo on immobilized 16 F **(D)**, and on immobilized AND-C4 **(E)**. **(F)** K_D_ values of 16 F and AND-C4 for rhEpo, rmEpo, and rhEV-3. n.b., no binding

Based on the transcriptional enrichment of hS3 in GBM tissue and cells, and on the lack of AND-C4 effect on CFU formation, we hypothesized EV-3 as the main target of AND-C4. To test this hypothesis, we first proceeded with the production of the EV-3 protein. It is worth reminding that, when compared to Epo, EV-3 lacks the region Δ27–55, including the AB loop (Fig. 5A), which corresponds to the EpoR-binding interface [55, 56]. After EV-3 purification from native protein extracts, Western blot images showed high purity (≥ 95%) of the final EV-3 glycoprotein (Fig. 5B).

The possible binding of AND-C4 to rhEV-3 was next evaluated through SPR. These analyses were carried out by evaluating the direct binding of rhEV-3, when flowing simultaneously on immobilized 16 F (as control, anti-Epo mAb) and AND-C4 (Fig. 5C). In parallel, the direct binding of rhEpo and of recombinant mouse erythropoietin (rmEpo) (Thermo Fisher Scientific, Waltham, MA, USA) were assessed. The results showed that 16 F bound rhEpo much better than rmEpo, but was unable to bind rhEV-3 (Fig. 5D). To the opposite, AND-C4 efficiently bound rhEV-3, while AND-C4 binding to rhEpo was poorly detectable, and to rmEpo was undetectable (Fig. 5E). The SPR results allowed us to quantitative evaluate the binding affinity of AND-C4 for rhEV-3 in comparison with rhEpo. As shown in Fig. 5F, the mean equilibrium dissociation constant (K_D_) for rhEV-3 was found to be 0.22 × 10^− 9^ M, a value found 100-fold lower than that for rhEpo (22.00 × 10^− 9^ M).

### EV-3 levels are high in human GBM tissue

**Fig. 6:**
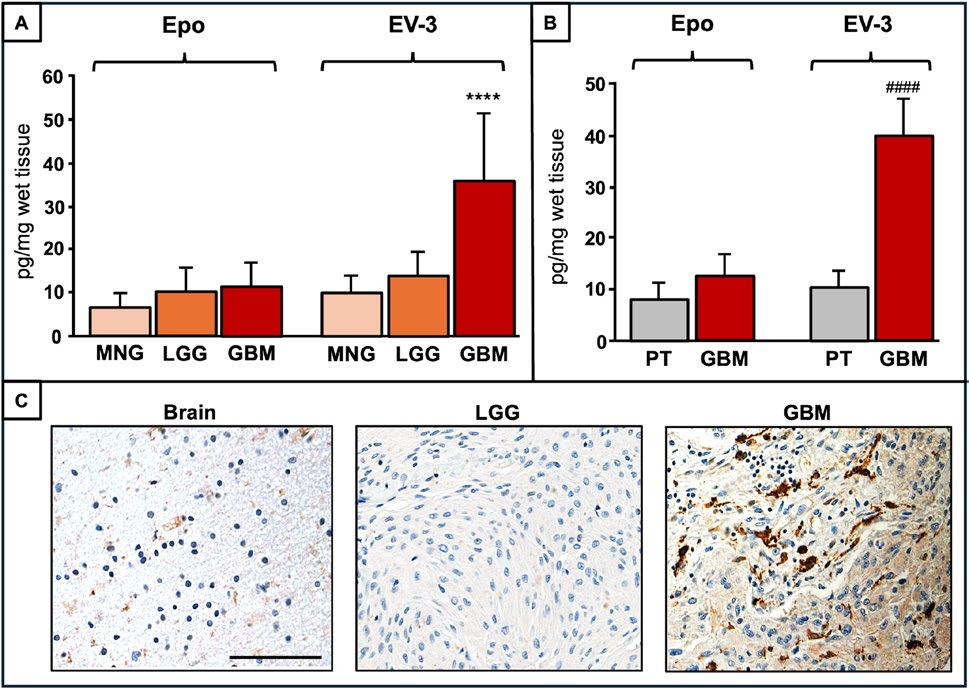
EV-3 content in different human brain tumors. **A **Content of Epo and EV-3 in human specimens from patients with meningioma (MNG) (*n* = 10), low grade gliomas (LGG) (*n* = 10) and GBM (*n* = 50). **B **Content of Epo and EV-3 in human GBM specimens (*n* = 10) and matched peritumoral tissue (PT) (*n* = 10). Each specimen was assayed in duplicate/triplicate. Data are expressed as mean ± SD. ****, *P* < 0.0001 vs. MNG; ####, *P* < 0.0001 vs. PT. **C **Representative immunohistochemical images stained with AND-C4 of human brain (Brain), LGG and GBM. Scale bar, 50 μm

In order to assess how much of the alternative splicing-generated variation observed at the hS3 mRNA level was present at the protein level, the levels of Epo and EV-3 were evaluated in a cohort of 70 tumor biopsies from patients affected by MNG, LGG, and GBM. Using specific ELISA for Epo and EV-3, we found that Epo levels were similar in the three types of brain tumors, whereas EV-3 content in GBM tissue was significantly higher (about 3-fold) than that in MNG and LGG (Fig. 6A). In addition, Epo values in GBM were similar to, whereas EV-3 levels were significantly higher (more than 3-fold) than those of cross-matched peritumoral brain (Fig. 6B). We found that the EV-3 content was in the range of peritumoral brain only in 2 out of 50 samples from GBM tissue (4%).

In order to validate the presence of the AND-C4 target in brain tumors, we evaluated its expression and distribution through IHC on tissue arrays comprising normal brain tissue, LGG, and GBM. As shown in the representative images of Fig. 6C, immunohistochemical analyses of human sections confirmed that AND-C4 target was undetectable in normal brain and LGG, but significantly expressed in GBM, yielding intense staining in different cells. GBM exhibited heterogeneous staining patterns, including a focal (40% of cases) or a diffuse (56% of cases) distribution. Overall, IHC analyses showed that the AND-C4 target was undetectable in sections from human brain and LGG, but expressed in the vast majority (96%) of GBM samples, confirming the ELISA data on high EV-3 levels in GBM.

### EV-3 is spatially distributed in human GBM tissue and enriched in hypoxic regions

**Fig. 7:**
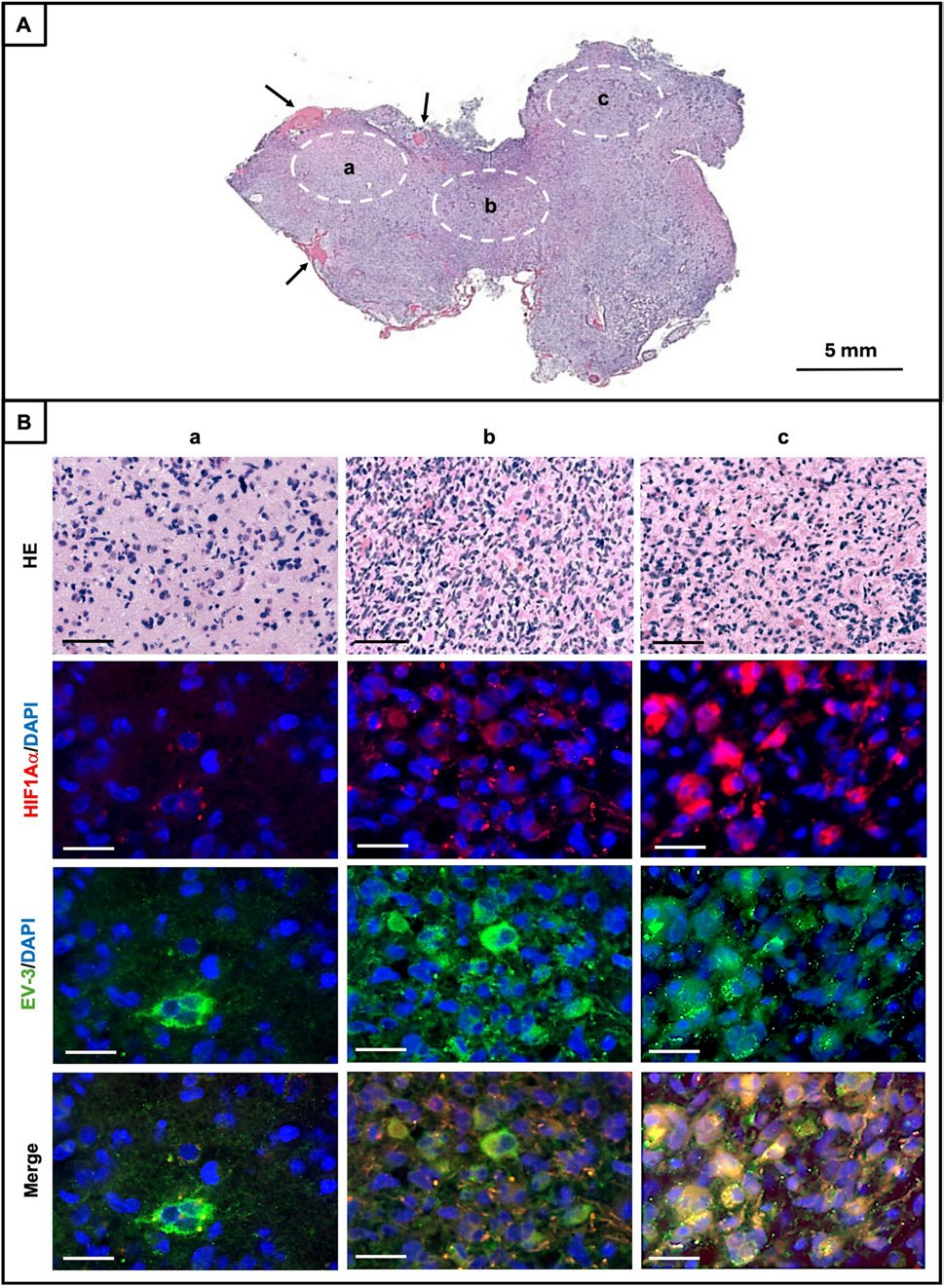
Spatial distribution of EV-3 and hypoxia marker HIF1α in GBM tissue. **A **H&E-stained section of a GBM biopsy showing three tumor regions (a-c) characterized by low (a), medium (b) and high (c) distance from blood vessels (black arrows). Dashed ellipses indicate the areas selected for immunofluorescence analyses. Magnification: 0.56x. **B **HE: Higher magnification (40x, scale bars: 50 μm) of H&E images from a-c regions illustrating local variations in tumor density and cellular morphology. Immunofluorescence images (60x, scale bars: 20 μm) of HIF1α (red), EV-3 (green), and nuclei (DAPI, blue) and merged images in the three indicated tumor regions

In addition, in order to determine the spatial distribution of EV-3 within the tumor microenvironment, we performed a double immunostaining for EV-3 and HIF1α on three different regions (called a-c) of a GBM specimen, characterized by different proximity/distance from blood vessels (Fig. 7A). The H&E staining of these regions revealed the typical heterogeneity of high-grade tumors, with regions of variable cellular density, marked nuclear pleomorphism, and disorganized architecture (HE in Fig. 7B).

Immunofluorescence analyses (Fig. 7B) showed that HIF1α, a well-established marker of hypoxia, exhibited a different distribution in the three regions, consistent with their vascular proximity. Indeed, it was undetectable in the well-vascularized region (a), but was present in the (b) region and highly abundant in the more hypoxic (c) region (Fig. 7B). HIF-immunofluorence was evident in cell nucleus and was abundant in the cell cytosol, indicating possible mechanistic interplay.

EV-3 immunofluorescence was detected in all three tumor regions, but with spatial heterogeneity. Indeed, signal intensity varied across analyzed regions, increasing from (a) to (c), (Fig. 7B), and reflecting its relation to tissue hypoxia. EV-3 immunofluorescence exhibited predominantly cytoplasmic localization. Notably, merged images (Fig. 7B, bottom) revealed high degrees of co-labeling between EV-3 and HIF1α in many cells (yellow color) particularly in regions exhibiting high distance from blood vessels that is hypoxic ones.

### EV-3 is efficiently produced and secreted by GBMCs and GSCs

**Fig. 8:**
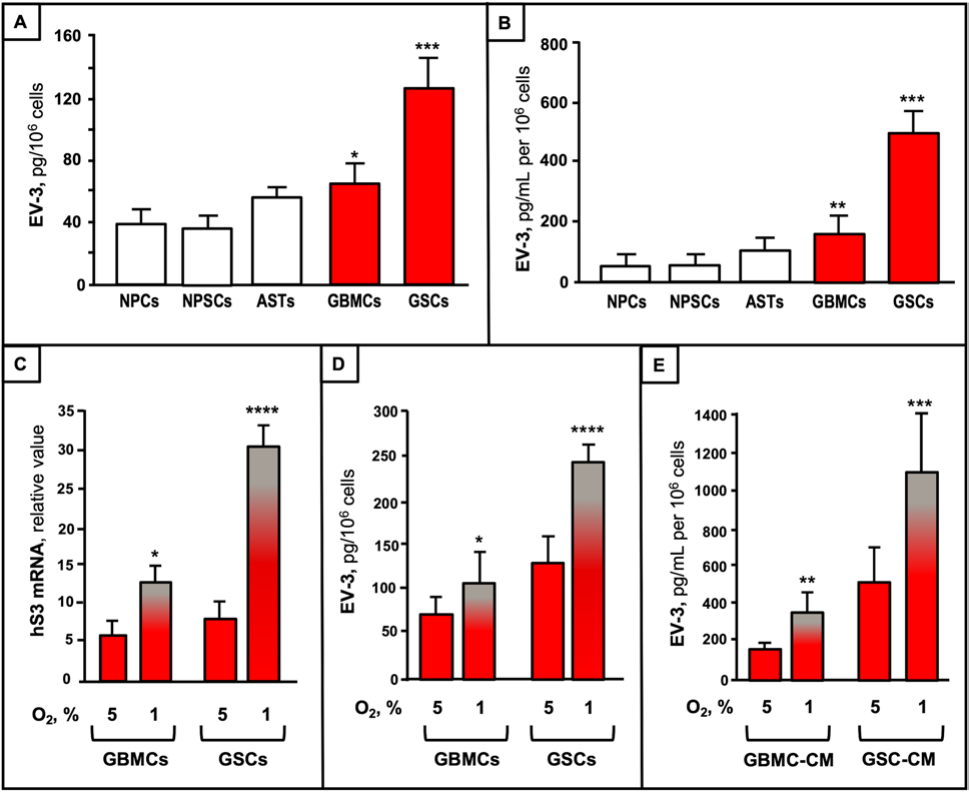
Cellular and extracellular levels of EV-3 in brain-derived and GBM-derived cells, and hypoxia effect. **A**,** B **Levels of cellular **(A)** and extracellular **(B)** EV-3 in NPCs ((*n* = 3), NPSCs (*n* = 3), ASTs (*n* = 3), GBMCs (*n* = 10), and GSCs (*n* = 10); **C**,** D **levels of hS3 transcript **(C)**, and EV-3 protein **(D)** in GBMCs (*n* = 10), and GSCs (*n* = 10) cultured under 5% or 1% O_2_ for 72 h. **E **EV-3 concentration in conditioned media (CM) from GBMCs and GSCs cultured under 5% or 1% O_2_ for 72 h. Data are the mean ± SD of triplicate quantifications. *, *P* < 0.05; **, *P* < 0.01; ***, *P* < 0.001; ****, *P* < 0.0001 vs. NPCs (in A and B), and vs. 5% O_2_ (in C-E)

To uncover the cellular origin of EV-3, we next evaluated EV-3 content in different cells from human brain and GBM tissue. The protein levels of EV-3 were found similar in NPCs, NPSCs, and ASTs, whereas both GBMCs and GSCs exhibited EV-3 values significantly higher than those of the different human brain cells (Fig. 8A). Notably, EV-3 levels in GSCs were found about 4-fold higher than those in human brain cells, reflecting its enhanced transcription, and revealing GSCs as crucial cells in EV-3 production. Further studies aimed at evaluating EV-3 as molecular component of the tumor microenvironment. Analyses of the conditioned medium from cells of human brain and GBM revealed that EV-3 was present in measurable amounts in all cases, with low levels in the conditioned media from normal brain cells, and a significant enrichment in both types of GBM-derived ones (Fig. 8B). EV-3 levels were significantly higher in GSC conditioned media than in GBMC ones (Fig. 8B), thus reflecting the variation observed in the cellular EV-3 content. Considering that hypoxia plays a pivotal role in neoplastic properties of GBM, including self-renewal of GSCs [57], we next investigated the effect of hypoxia on transcript expression and protein levels in GBMCs, GSCs and their conditioned media. Interestingly, under hypoxic conditions (O_2_, 1%), the levels of both hS3 mRNA, and EV-3 protein were significantly increased in GBMCs, and particularly in GSCs (Fig. 8C, D). At low O_2_ levels, EV-3 markedly increased not only in tumor cells, but also in their conditioned media, with GSCs exhibiting the highest extracellular levels again (Fig. 8E).

### EV-3 acts as oncogenic protein in human GBM cells and AND-C4 antagonizes its action

**Fig. 9:**
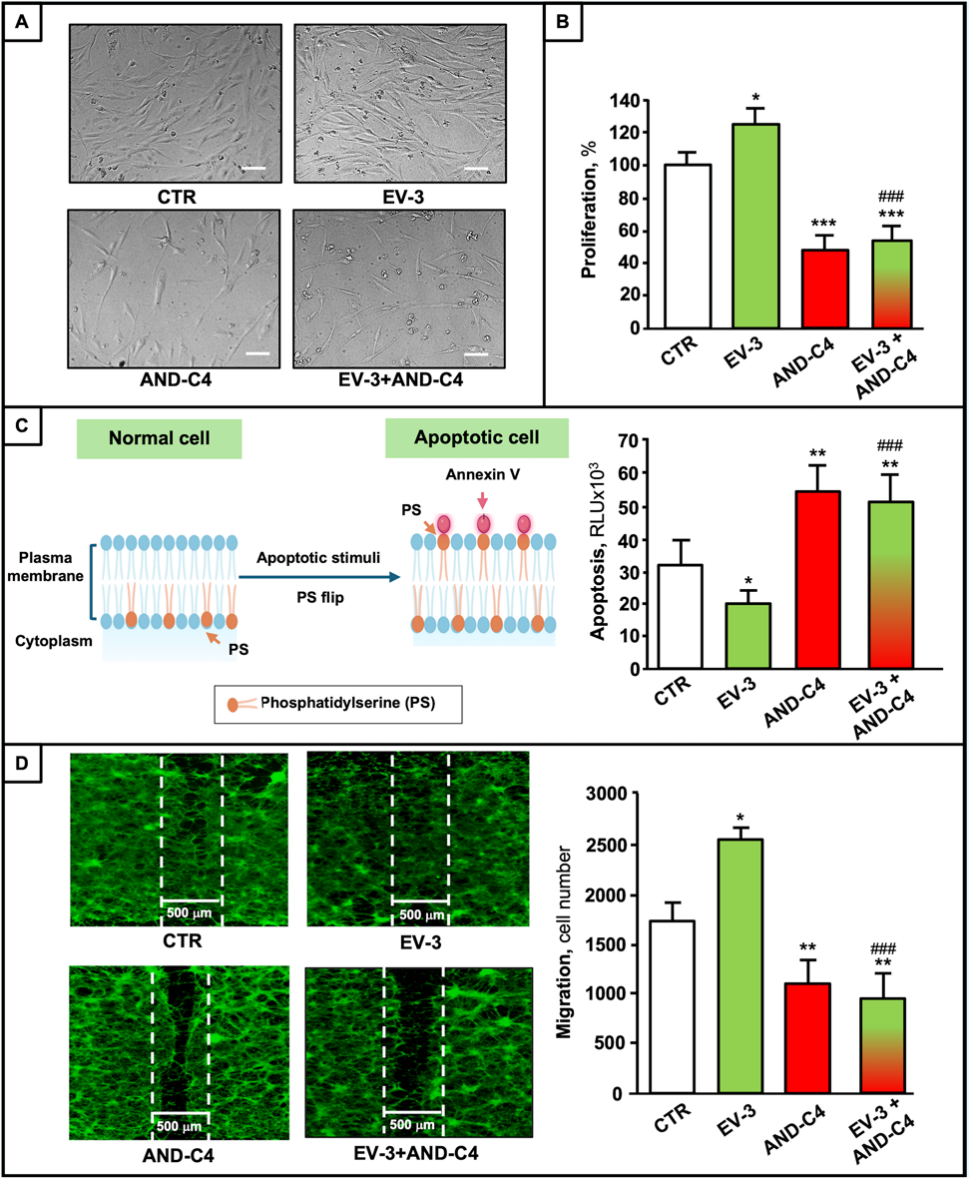
Effects of EV-3 and AND-C4 on oncogenic properties of GBMCs. GBMCs were treated with rhEV-3 (100 ng/mL) or/and AND-C4 (10 µg/mL) for 48–96 h. **A **Representative microscopic images of GBMCs (scale bar 100 μm), and **(B)** effects of different treatments (for 96 h) on GBMC proliferation. **C **Schematic representation of Annexin V apoptosis assay (left), and effects of different treatments (for 96 h) on GBMC apoptosis (right). **D **Representative fluorescence microscopic images of GBMCs stained with calcein after migration assays (left), and quantification of GBMC migration after different treatments for 48 h (right). All data are reported as mean ± SD, with each sample run in triplicate. *, *P* < 0.05, **, *P* < 0.01 and ***, *P* < 0.001 vs. control, untreated cells (CTR). ##, *P* < 0.01 and ###, *P* < 0.001 vs. EV-3

We next investigated the effect of EV-3 and AND-C4, as well as their combination, on brain cell lines and GBM-derived cells. As shown in the representative microphotographs of GBMCs, EV-3 did not affect GBMC morphology, whereas AND-C4 treatment resulted in a pronounced cell shrinkage, membrane blebbing, and detachment of these cells (Fig. 9A). We found that rhEV-3 and AND-C4-induced significant, but opposite effects on GBMC proliferation (Fig. 9B), and apoptosis (Fig. 9C), rhEV-3 acting as stimulator, and AND-C4 as inhibitor of both processes. When rhEV-3 and AND-C4 were combined, the inhibitory effects of the mAb dominated. To the contrary, neither rhEV-3 nor AND-C4 affect proliferation and apoptosis of human brain cell lines, including NPCs, NPSCs, and ASTs (Fig. S1). Moreover, EV-3 was able to significantly promote the migration rate of GBMCs, and AND-C4 completely inhibited it both in basal conditions, and even after EV-3-stimulation (Fig. 9D).

### rhEV-3 promotes the proliferative, anti-apoptotic and stemness properties of GSCs, and AND-C4 antagonizes all these effects

**Fig. 10:**
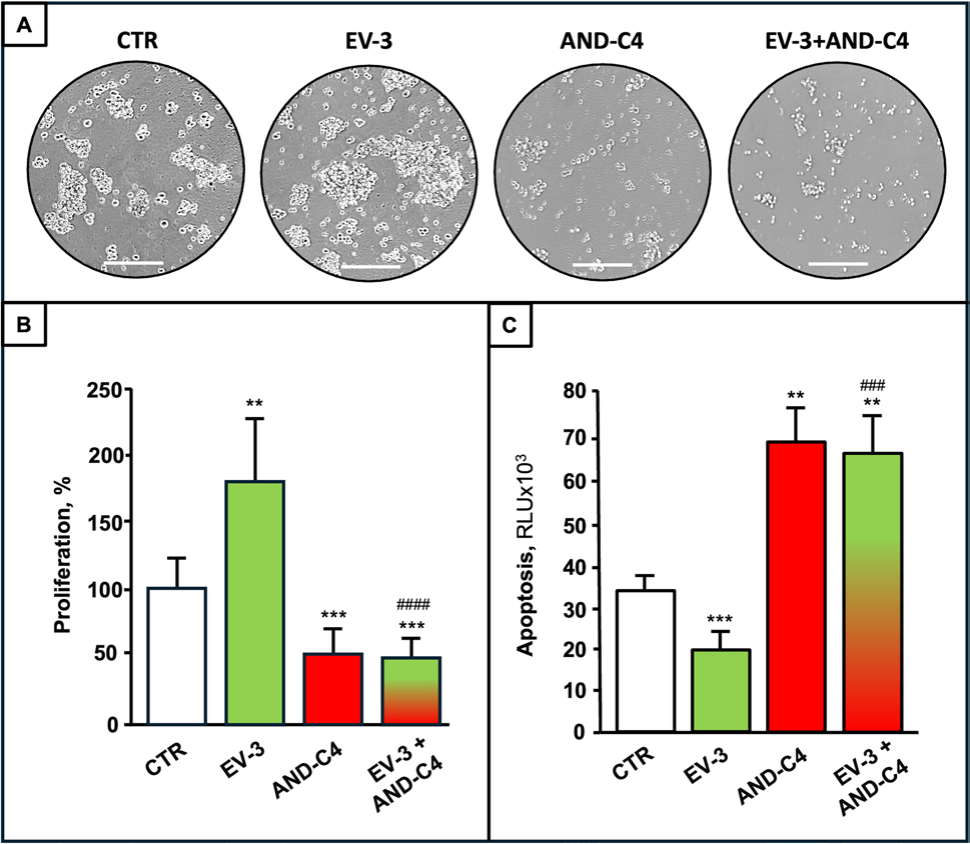
Effects of EV-3 and AND-C4 on the oncogenic properties of GSCs. rhEV-3 (100 ng/mL) and AND-C4 (10 µg/mL), alone or in combination, were administered to GSCs (*n* = 10) for 96 h. **A **Representative microscopic images (scale bar, 100 μm) of GSCs at the end of different treatments. **B** and **C **Effects of different treatments on GSC proliferation **(B)** and apoptosis **(C)**. Data are the mean ± SD, with each sample run in triplicate. CTR, control cells. **, *P* < 0.01, and ***, *P* < 0.001 vs. CTR; ###, *P* < 0.001, and ####, *P* < 0.0001 vs. EV-3 treated cells

As in the case of GBMCs, EV-3 treatment prompted GSC proliferation, which was significantly reduced by AND-C4 (Fig. 10A, B). Furthermore, EV-3 administration was found inhibit GSC apoptosis, whereas AND-C4 significantly fostered it, either in the absence or presence of EV-3 (Fig. 10C).

**Fig. 11:**
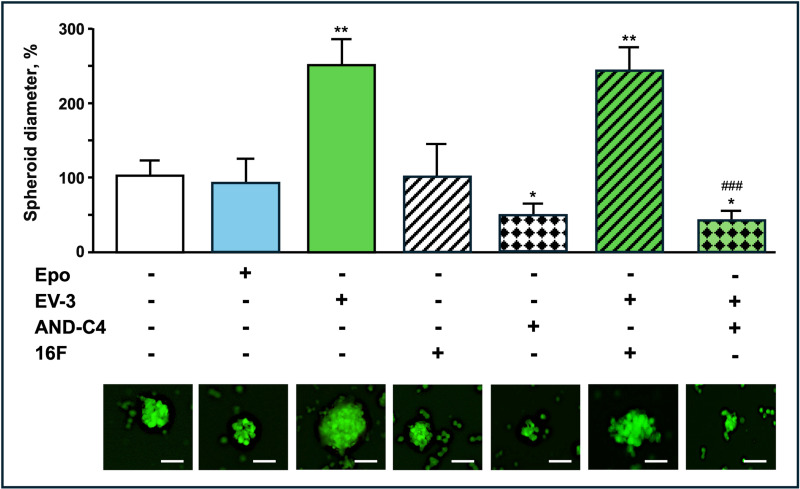
Effects of Epo, EV-3 and AND-C4 on GSC spheroid formation. GSCs were incubated with rhEpo (10 IU/mL), rhEV-3 (100 ng/mL), 16 F (10 µg/mL), and AND-C4 (10 µg/mL), alone or in combination, for 96 h. Upper part; spheroid diameter of GSCs (up), and representative fluorescence images of GSC spheroids (down) stained with calcein AM (scale bar, 100 μm). Data are the mean ± SD of triplicate assays. *, *P* < 0.05 and **, *P* < 0.01 vs. control cells. ###, *P* < 0.001 vs. EV-3 treated cells

We then investigated the effects of EV-3 and of AND-C4 on neurosphere formation of GSCs, a defining feature of GSCs, reflecting their self-renewal and tumorigenic potential. As shown in Fig. 11, in the used culture conditions, control cells robustly formed neurospheres of typical size and morphology, and rhEV-3 treatment significantly enhanced this formation. Indeed, after GSCs labelling with calcein as a vital dye, fluorescence microscopy images revealed that rhEV-3 promoted the generation of spheroids with a diameter larger (∼2.5-fold) than that of controls. Noteworthy, in contrast to the neurosphere promoting effect of rhEV-3, similar doses rhEpo were without effects (Fig. 10).

AND-C4 treatment markedly reduced neurosphere formation, with cell aggregates exhibiting non-viable cells, and a debris-filled morphology with the presence of adherent flattened cells, rather than cohesive, spherical structures (Fig. 10). Co-treatment of rhEV-3 with AND-C4 not only abrogated the rhEV-3-driven expansion but also induced a widespread disintegration of neurospheres (Fig. 11). The administration of the anti-Epo Ab 16 F, either alone or in the presence of EV-3, was ineffective on neurosphere formation.

### rhEV-3 promotes tumor angiogenesis and inhibits immune cell migration, with AND-C4 inhibiting both processes

In order to further dissect the role of rhEV-3 and AND-C4 in the GBM microenvironment, GECs and immune cells (as PBMCs) were treated with these molecules, and cell response was recorded.

**Fig. 12:**
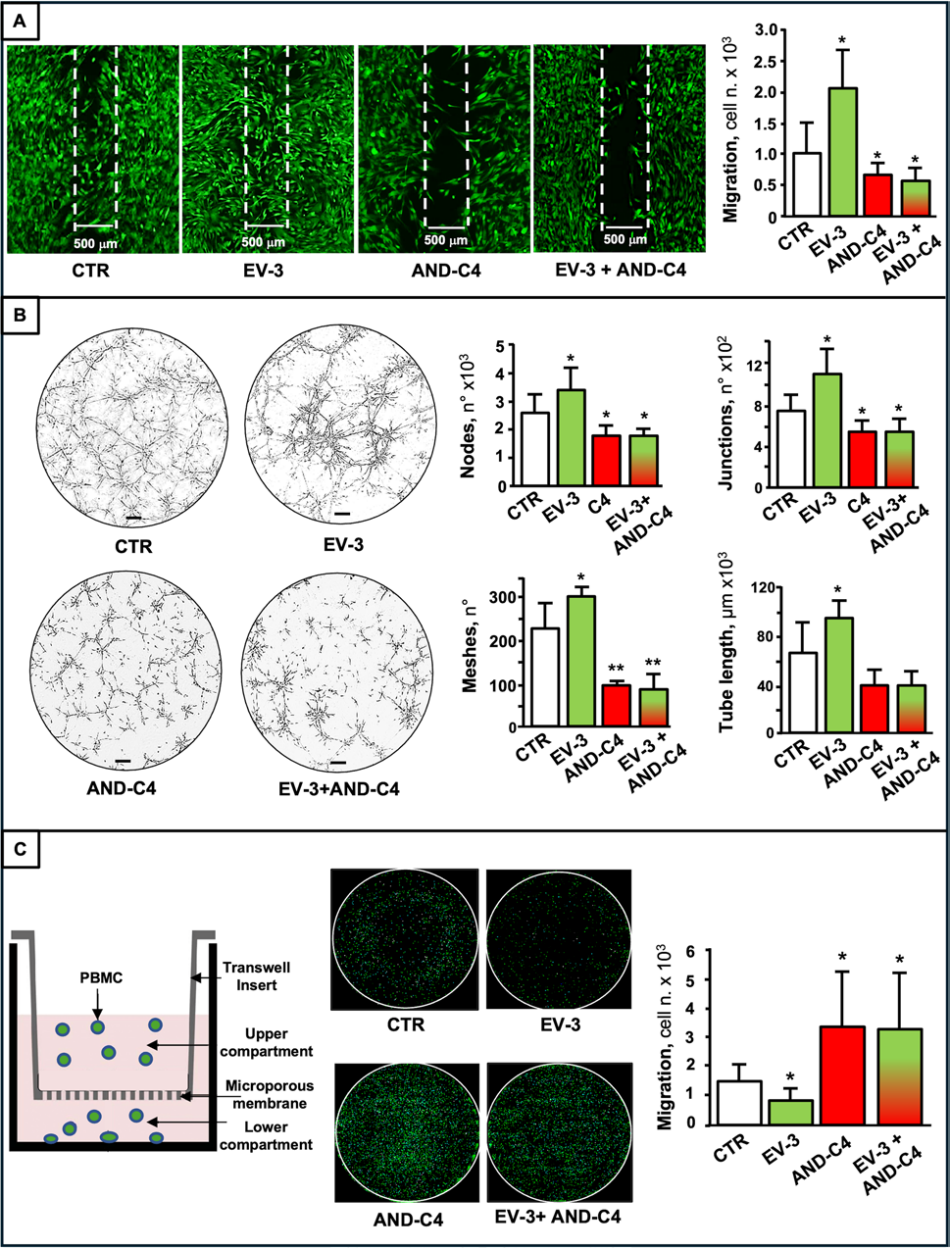
Effects of EV-3 and AND-C4 on angiogenesis and immune cell migration. **A** and **B **GBM-derived endothelial cells (GECs) were treated with rhEV-3 (100 ng/mL), or AND-C4 (C4, 10 µg/mL), or their combination for 48 h. **A **Representative fluorescence microscopic images of GECs stained with calcein (left), and results of cell migration (right) at the end of the migration assay. **B **Representative microscopic images (scale bar, 100 μm) (left) and related quantification of angiogenesis parameters (right) of GECs after different treatments. **C **Left, schematic representation of the modified Boyden chamber used to assess PBMC migration. Middle, representative images of calcein-stained PBMCs. Right, number of PBMC migrated in the lower compartment containing rhEV-3 (100 ng/mL) and/or AND-C4 (10 µg/mL). All data are the mean ± SD of triplicate assays. *, *P* < 0.05, and **, *P* < 0.01 vs. control cells (CTR)

After treatment with rhEV-3, GECs exhibited a markedly increased migration rate (Fig. 12A), as well as enhanced angiogenesis (assessed as formation of nodes, meshes, junctions and tubes) (Fig. 12B). In contrast, AND-C4 alone significantly inhibited both GEC chemotaxis and angiogenic sprouting, and, after co-treatment with rhEV-3, it fully abrogated its pro-angiogenic effect (Figs. 12A, B). In addition, immune cell assays revealed that rhEV-3 reduced chemotaxis of peripheral blood mononuclear cells (PBMCs), whereas AND-C4 significantly enhanced migration response of PBMCs toward the tumor milieu, even in the presence of rhEV-3 (Fig. 12C). In the used experimental conditions, AND-C4 was without significant effect on proliferation and apoptosis of both GECs and PBMCs (Fig. S2).

### In vivo studies: pharmacokinetics, and systemic effects of AND-C4 in nude mice

**Fig. 13:**
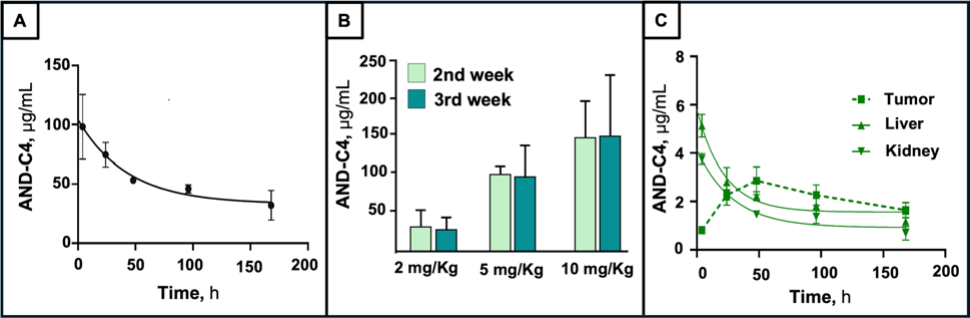
Pharmacokinetics of AND-C4 in mice. Nude mice were i.v. treated with AND-C4 (2–10 mg/Kg) for different times. **A **Levels of AND-C4 in plasma from mice i.v. treated with AND-C4 (2 mg/Kg) for different times. Each value is the mean ± SD of 3 animals. **B **Plasma levels of AND-C4 in mice after i.v. treatment with different doses of AND-C4 for 2 and 3 weeks. Each value is the mean ± SD of 3 mice. **C **Pharmacokinetic profile of AND-C4 showing the levels of AND-C4 in tumor, liver and kidney from mice treated with AND-C4 (2 mg/Kg) for different times. Data are the mean ± SD of 3 mice

Prompted by the anti-tumoral effects of AND-C4 in cultured cells, we next proceeded with in vivo studies. First, we evaluated the tolerability, toxicity and pharmacokinetics of the mAb in nude mice. After intravenous injection of AND-C4 at different doses (up to 10 mg/kg) for different times (up to 7 weeks), animal body weight remained unchanged, and neither acute clinical signs of disease, nor clinical/behavioral abnormalities were observed (data not shown). During treatment, AND-C4 pharmacokinetics was evaluated in blood, liver, kidneys and tumor by SPR. After intravenous injection of 2 mg/Kg of the mAb, the sensorgrams revealed that the highest plasma level of AND-C4 was found at the earliest investigated time (one hour after treatment), and this level gradually declined with time, overall exhibiting a half-life of ∼ 4 days (Fig. 13A). In addition, after administration of increasing doses of AND-C4, a dose-dependent elevation of plasma mAb levels was evident after 2 and 3 weeks of treatment (Fig. 13B). In liver and kidney, the time-course profiles of AND-C4 were similar to that of blood, but the mAb levels were found 3-4-fold lower than those of plasma (Fig. 13C). Different was the kinetics of tumoral AND-C4, its levels increasing in the first 2 days after treatment and remaining nearly constant thereafter (Fig. 13C). In the used experimental conditions, no relevant effect of AND-C4 treatment was recorded on red blood cell (RBC) counts, hematological parameters, and white blood cells (WBC) (Fig. S3), as well as on different blood markers of kidney and liver function (urea, creatinine, albumin, ALT and AST) (Fig. S4). These findings underscore that, in the used dose range (2–20 mg/kg), AND-C4 does not exhibit either hematological or systemic toxicity.

### In vivo studies: AND-C4 exerts anti-tumoral effects in a heterotopic mouse model of GBM

**Fig. 14:**
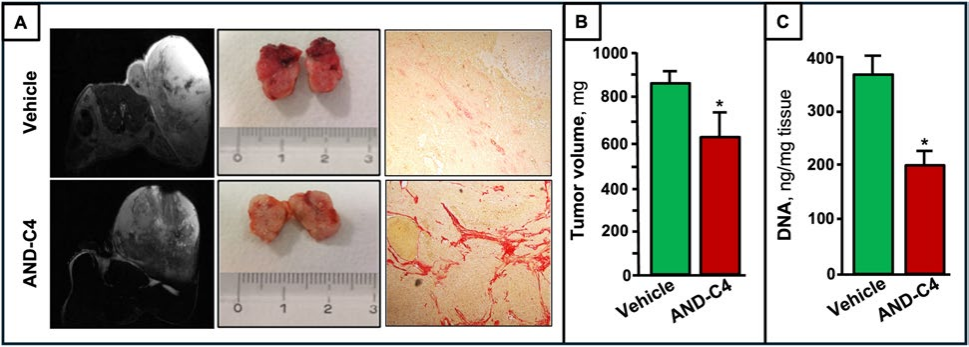
Antitumor effects of AND-C4 in a heterotopic GBM mouse model. **A**,** left **Representative MRI images, **A**,** middle **macroscopic appearance, and **A**,** right** microscopic images for Sirius Red staining of PDX tumors from mice treated with vehicle or AND-C4 (10 mg/Kg). **B and C **Tumor volume and tumor DNA content, respectively, of mice i.v. treated with vehicle or AND-C4. Data are the mean ± SD. *, *P* < 0.05, vs. vehicle-treated mice

The PDX model (involving direct implantation of biopsied tumor tissue into flank subcutaneous space of immune-deficient animals) was first chosen as model for practicality reasons (e.g., technical feasibility and easy follow-up of tumor formation). This model was used to evaluate AND-C4: (1) effect on body mass; (2) ability to pass the blood-brain barrier (BBB); and (3) effect on tumor growth. This model was not used to evaluate the mAb effect on animal survival as, to avoid animal pain and distress, mice were sacrificed when tumor volume was higher than 2000 mg (or clinical/behavioral abnormalities appeared). MRI images of PDX-derived tumors showed that tumor volume was similar in control and AND-C4 treated mice, but a different gross composition was present. In fact, image analysis of T2 sequences at day 17 post treatment revealed that tumors of AND-C4-treated animals were characterized by a significant enrichment of hypo-intense fibrotic tissue (Fig. 14A, left). At the end of the experiment, the tumor macroscopic appearance of AND-C4-treated mice was much more compact than that from controls (Fig. 14A, middle). Moreover, histochemical examination after Sirius Red staining for collagen of tumors from AND-C4-treated (but not of controls) mice revealed the presence of intense positive areas at the tumor periphery, with fibrillary branches penetrating the tumor mass (Fig. 14A, right), supporting MRI findings. Finally, we found that the tumor effective volume (Fig. 14B), and its content of DNA (as cellularity index) (Fig. 14C) from AND-C4 treated animals were both significantly lower than those from vehicle-treated mice. Altogether these findings supported that AND-C4 was able to exert in vivo anti-tumoral effect in a heterotopic GBM model.

### In vivo studies: AND-C4 inhibits tumor growth and increases animal survival in an orthotopic mouse model of GBM

Lastly, we investigated the potential effects of intranasally administered AND-C4 on an orthotopic GBM model. The intranasal administration is a non-invasive method that bypass BBB restrictiveness, thus providing rapid drug absorption and higher local concentrations at the tumor site [58, 59].

**Fig. 15:**
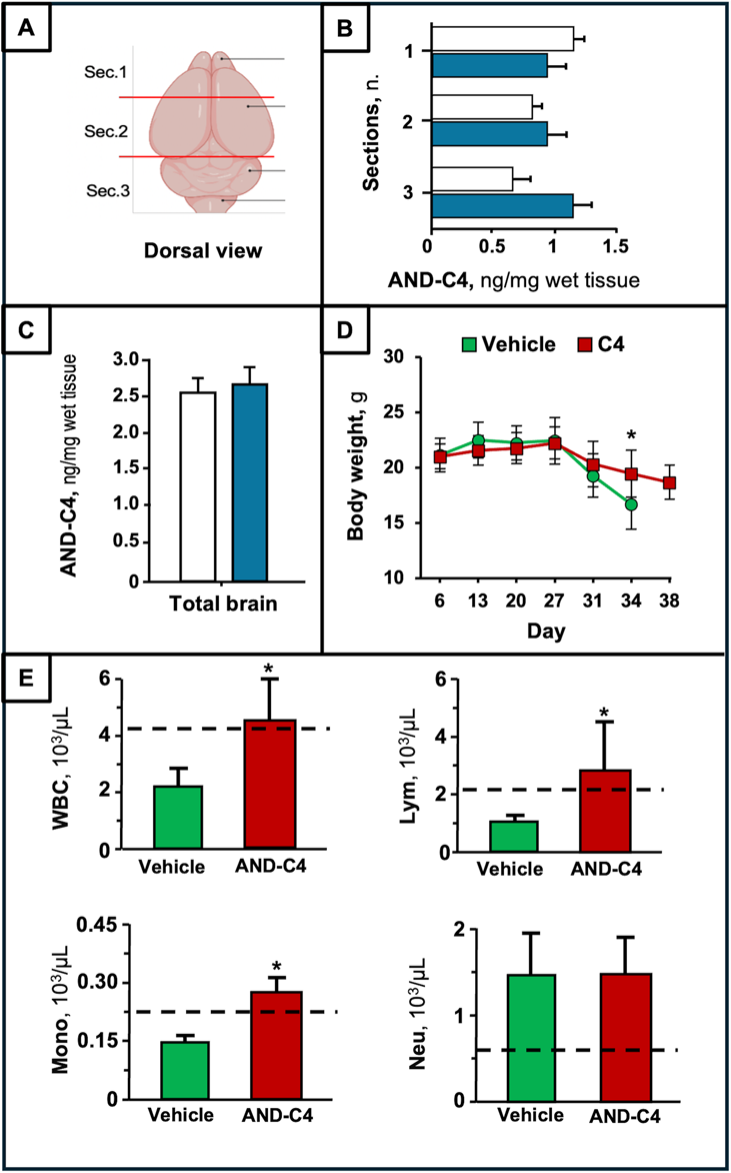
Biodistribution and systemic effects of intranasally administered AND-C4 in the orthotopic GBM mouse model. **A **Illustration of the radial sections (Sec.) used for mice brain sampling after sacrifice. AND-C4 levels **(B)** in different brain sections and **(C)** in total brain from mice intranasally treated with AND-C4 for 3 (white histograms) and 6 (blue histograms) hours. **D **Body weight at different treatment times in vehicle and AND-C4-treated mice. *, *P* < 0.05 vs. vehicle. **E **Number of white blood cell (WBC), lymphocytes (Lym), monocytes (Mono), and neutrophils (Neu) in blood from controls (green) and AND-C4 (red) mice. Dotted lines represent lower physiological limit. Upper limits (10^3^/µL) are 16.2 for WBC, 11.3 for Lym, 1.5 for Mono, and 4.31 for Neu. Data are mean ± SD. *, *P* < 0.05 vs. vehicle

In order to evaluate that AND-C4 after intranasal administration can reach the brain, we first performed a biodistribution study. The results revealed that, 3 and 6 h after intranasal administration, AND-C4 was able to reach different brain regions, assessed as three parallel sections (Fig. 15A). At the earliest investigated time (3 h from treatment), the highest AND-C4 levels were found in the frontal brain (Sect. 1), where the olfactory bulb resides, and its values were less than half in Sects. 2 and 3 (Fig. 15B). After 6 h from treatment, AND-C4 decreased in Sect. 1, remained nearly constant in most of the cerebrum (Sect. 2), but exhibited a marked increase in the posterior part (Sect. 3) (Fig. 15B). These results reflect a rapid biodistribution of AND-C4 from the anterior to the posterior brain region. In addition, AND-C4 concentration in the total brain was found similar at the two investigated times (Fig. 15C), supporting a constant brain content in this time lapse.

In mice with orthotopic GBM, the analysis of body weight dynamics showed that animal weight remained constant until day 27 post implantation, decreasing thereafter in both experimental groups (vehicle and AND-C4-treated), and particularly in the control group (Fig. 15D). The data showed that AND-C4-immunized mice were protected from excessive weight loss starting from day 31 after tumor implantation. At day 34, the mean body weight of AND-C4-treated animals was significantly higher than that of controls. At day 38, when all controls were dead, the body weight of AND-C4-treated mice was similar to that of controls at day 31 (Fig. 15D), reflecting a marked delay in weight loss.

Hematological analyses demonstrated that RBCs and related parameters were similar in the two experimental groups, indicating that AND-C4 treatment did not influence either RBC number, or their features (Fig. S5). To the opposite, a significant increase of total white blood cells (WBC), was observed in the AND-C4 treated group with respect to the control one (Fig. 15E). This variation was found mainly consequent to a significant increase (more than 3-fold) of lymphocyte number, which was found about one-half the lower physiological limit of mice.

**Fig. 16:**
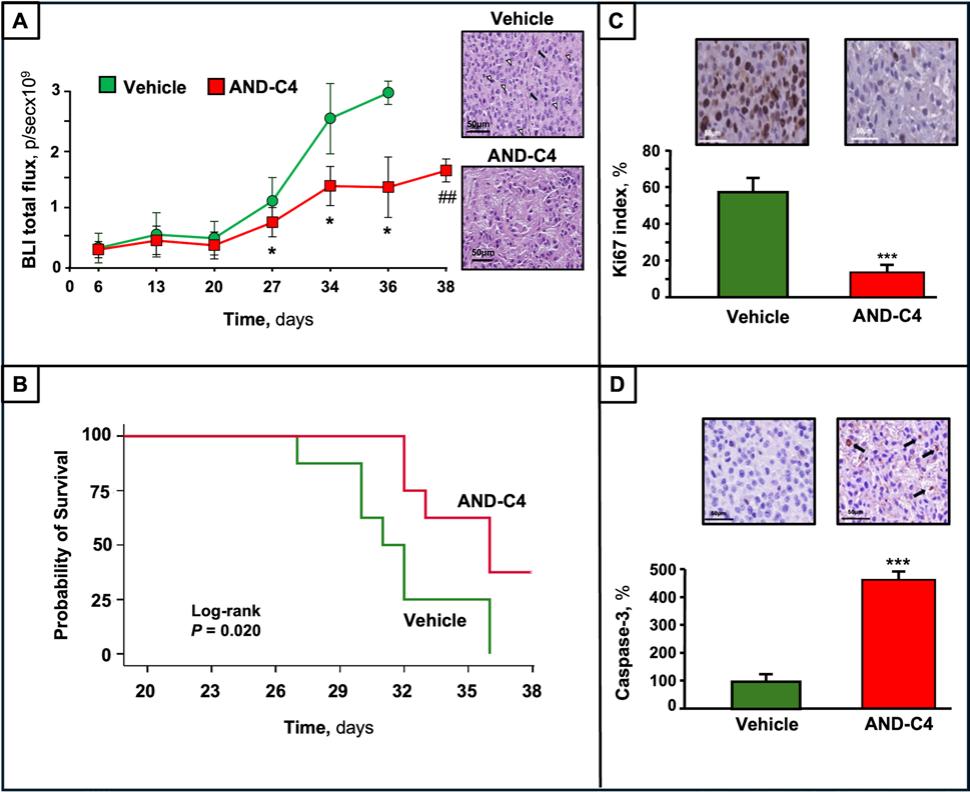
Anti-tumor effects of intranasally administered AND-C4 in the orthotopic GBM mouse model. **A **Graphic representation of BLI total flux along with time. *, *P* < 0.05 vs. vehicle at the same day; ##, *P* < 0.01 vs. vehicle at day 36, with representative H&E images (scale bar, 50 μm) of brain tumors from control (upper) and AND-C4-treated (lower) animals. Atypical cells with numerous mitotic figures (white arrowheads), and numerous blood vessels (black arrows) were present in the tumor mass of controls but not in that of AND-C4-treated mice. **B **Kaplan Maier survival curves of mice in vehicle and AND-C4-treated groups. Log-rank test was used for group comparison. **C** Representative Ki-67 immunostaining images (scale bar, 50 μm) of brain tumors from control and AND-C4-treated mice, and corresponding values of Ki-67 index. ***, *P* < 0.001 vs. vehicle. **D **Representative caspase-3 immunostaining images (scale bar, 50 μm) of brain tumors from vehicle, and AND-C4 treated animals and corresponding values of immune-stained area of caspase-3. ***, *P* < 0.001 vs. vehicle

BLI data in control mice showed that tumor volume started to increase on day 27 after tumor implantation, and this increase proceeded thereafter (Fig. 16A). AND-C4 treatment was followed by a potent effect on tumor volume, i.e. by a significant reduction of tumor growth along with time (Fig. 16A). Histological analyses of H&E-stained sections of GBM revealed that AND-C4-treatment was able to reduce either cellular density or nuclear atypia, as well as tumor vascularization and mitotic activity, and these features were associated with abundant fibrillary elements (Fig. 16A).

Analyzing animal survival, at day 32 after tumor implantation, 25% of mice were alive in the control group and 75% in the AND-C4-treated one. At day 38 from tumor implant (the experimental end point), there was no survivor among the control mice, whereas 38% AND-C4-treated mice were alive. A Kaplan-Meier survival curve (Fig. 16B) showed a significant increase in survival (*P* = 0.020; log-rank test) of mice that had undergone AND-C4 intranasal treatment to those mice treated with placebo. These results demonstrated the efficacy of experimental intranasal administration of AND-C4 in brain-tumor bearing nude mice, and that AND-C4 exerts a survival promoting effect in mice with orthotopic GBM.

In addition, Ki-67 (a proliferative index) was high (more than 50%) in tumors from controls. and markedly reduced (to less than 20%) in those from AND-C4 treated mice (Fig. 16C). Finally, when cell apoptotic rate was assessed by caspase-3 staining, tumors in AND-C4-treated mice showed a significant increase in the number of apoptotic cells with respect to vehicle-treated ones (Fig. 16D).

## Discussion

GBM remains one of the most devastating and therapeutically resistant malignancies, primarily due to its intrinsic heterogeneity, and the presence of a unique, dynamic microenvironment with GSCs affecting and dictating its malignant properties. Focusing on these GBM features, this translational research enriches our knowledge on GBM microenvironment, identifying for the first time EV-3 (an Epo-V) as a key actor in GBM malignant features, with its targeting exhibiting promising therapeutic effects.

Our study started with RNA transcript investigations, focusing on Epo-Vs. Previous studies reported that *EPO* mRNA was overexpressed in GBM as compared to human brain [60]. As the *EPO* gene codes for different transcriptional variants [28], we here explored the presence of *EPO* transcript variations in GBM. It was of relevance to find a significant elevation of some *EPO* transcripts, namely *EPO*, hS3, hS4, and h1-4. As *EPO* expression includes all these transcriptional variants, the finding of *EPO* mRNA elevation does not appear to reflect the enrichment of the specific *EPO* transcript, but rather the sum of hS3, hS4, and h1-4 mRNAs. Notably, this increase of Epo-V transcripts was found in human GBM tissues when compared to both human brain and matched peritumoral tissues, as well as in primary, patient-derived cells when compared to brain ones, with GSCs exhibiting the highest increase. Of relevance, the hS3 transcript, which is translated into the EV-3 protein, was the most abundant *EPO*-derived mRNA, significantly overexpressed in GBM tissues, and their derived GBMCs, and especially GSCs.

On these premises and considering the great heterogeneity of both *EPO*-derived proteins and Epo receptors, we developed anti-Epo mAbs, and accurately proceed through a selection on the bases of different functional properties, including absence of erythropoietic effects, lack of toxicity on human brain cells, and potent anti-tumor actions. A distinguishing aspect of this selection was the set up and use of patient-specific functional screening platform, the Gliosphera. This platform enabled us to simultaneously profile the effects of different clones of AND-mAbs, capturing differential sensitivity of both human brain cells and patient-derived tumor cells, and providing a comprehensive readout of mAb activity. The Gliosphera assay led us to finally identify and select AND-C4 as the lead mAb with potent anti-tumoral effects, and deprived not only of erythropoietic actions, but also of toxicity on different human brain cell lines.

SPR analyses demonstrated that AND-C4 mAb exhibited an overall high affinity for rhEV-3 with K_D_ values in the sub-nanomolar range, and this value was found one-hundred lower than that for rhEpo. The selection and characterization of AND-C4 was fundamental for the progression of our investigation, as the mAb was then used either as a tool for the set-up of a custom ELISA, or for the identification of its target, or for testing its potential as therapeutic agent.

Using the Gliosphera platform, we demonstrated that AND-C4 efficiently reduced, in a dose-dependent fashion, proliferative and anti-apoptotic features of GBMC and GSC, and abolished the proliferative and anti-apoptotic effects of rhEV-3. Moreover, and notably, in neurosphere assays, AND-C4 potently inhibited GSC self-renewal, and triggered apoptosis, even in the presence of exogenous EV-3.

A relevant, novel finding of our study was the discovery of EV-3 as the Epo-V significantly enriched in patient-derived GBM tissue when compared to both human brain and peritumoral tissue, but not in other intracranial tumors, including MNGs and LGGs. Opposite to increased EV-3, we found Epo levels in GBM tissue similar to other brain tumors, as well as to cross-matched peritumoral tissue. A recent study reported that GBM cyst fluid contained different hormones, including Epo [16]. Despite tumor volume did not correlate with cyst fluid concentration of Epo, patient survival inversely correlated with cyst Epo levels, and these levels were higher than in serum, leading the Authors to suggest Epo formation by tumor or brain tissue. Our results do not appear to confirm this hypothesis. Although we cannot exclude that Epo from tumor, brain or blood might selectively accumulate in GBM cyst fluid, it is reasonable to assume that EV-3, more than, or together with, Epo is enriched in GBM cyst fluid.

In human GBM specimens, EV-3 was markedly (about 4-fold) and significantly elevated in tumor versus peritumoral tissues, suggesting its potential as diagnostic marker. A further clinical validation of the selective AND-C4 target expression in GBM was obtained by IHC staining, which confirmed the absence/low presence of AND-C4 target in normal brain and LGG, opposite to its abundancy in patient-derived GBM tissues. These findings strongly suggest a promising role of AND-C4 as therapeutic target for GBM.

Considering recent findings highlighting significant spatial heterogeneity within GBM tumors as a key factor contributing to its malignant features [61], in the next step, we spatially analyzed EV-3 densities in relation to tumor heterogeneity. With the used imaging modalities, we first found HIF1α enrichment in hypoxic areas, thus confirming its key role in mediating cellular adaptation to hypoxia. Intriguingly we found that in GBM cells the HIF1α protein localized not only in the nucleus (as expected) but also, and abundantly, in the cytosolic region. A possible interpretation of this finding may result from recent literature revealing that pulses in nuclear levels of HIF1α occur in cancer cells, the transient nature of nuclear HIF1α being required to avoid cell death [62, 63]. Notably, a dynamic intracellular trafficking of HIF1α emerged as essential not only for its nuclear transcription modulation, but also for its favoring translation of hypoxia-responsive proteins in the cytoplasm [64].

Of relevance, our spatial immunofluorescence staining allowed for the identification of spatial heterogeneity of EV-3 protein in human GBM, with hypoxic tumor areas as highly enriched ones. Our detection of EV-3-rich niches in hypoxic zones of GBM offers a novel, nuanced understanding of spatial heterogeneity of this cancer.

In further investigation, we found that EV-3 protein was present in low amounts in cultured human brain cell lines, including neural stem cells, neurons, and astrocytes, and it was highly increased in GBM patient-derived primary cell cultures, and particularly in GSCs. We thus found a correspondence between the alternative splicing-generated increase observed at the hS3 mRNA and the encoded protein EV-3 level. Thus, an *EPO* alternative splicing pattern emerged as a characteristic of GBM tissue and cells, fortifying the knowledge that alternative splicing is profoundly involved in cancer progression [23, 65]. In GBM, changes in splicing isoforms can alter the expression of the corresponding proteins and promote the generation of malignant phenotype [25, 26, 66, 67]. At present the mechanisms underlying dysregulation of *EPO* pre-mRNA alternative splicing in GBM, such as abnormal expression, mutation, or post-translational modifications of splicing factors [23, 66], remain unknown. Our study prompts future research on this direction.

It is worth noting that in our study GSCs emerged as the most efficient cells in expressing, producing and, not last, secreting EV-3, and these processes were potentiated in hypoxic conditions. Hypoxia characterizes GBM, and is an environmental cue essential to the presence and self-renewal of GSCs [57, 67–69]. Indeed, the reduction of oxygen levels plays a key role as an effective inducer of GBM adaptation and survival, by regulating GSC proliferation, trans-differentiation, and angiogenesis [70, 71]. Hypoxia determines the upregulation of HIFs, which in turn boost the transcription of onco-promoter mediators, including the *EPO* mRNA [71, 72]. HIF1α, a recognized master regulator of *EPO* expression, has been linked with increased GBM invasiveness and angiogenesis, thus contributing to GBM aggressive behavior and highly angiogenic nature [73]. Considering our immunofluorescence analyses, showing significant EV-3 abundance in hypoxic, HIF1α-enriched GBM regions, and previous studies demonstrating increased HIF1α in GBMCs and GSCs under hypoxia [70, 73, 74], it emerges that the hypoxic microenvironment characterizing GBM favors HIF1α, resulting in EV-3 up-regulation.

Further results of our study showed that the addition of rhEV-3 to primary patient-derived GBM cells (GBMCs and GSCs) stimulated their proliferation, invasion, anti-apoptotic action, angiogenesis and stemness, identifying EV-3 as a novel mediator and driver of key oncogenic properties of GBM. We thus propose an innovator regulatory mechanism for GBM malignancy in which EV-3 from human GBM, and particularly from GSCs, acts in an autocrine/paracrine fashion to promote malignant cell properties. The reliability and robust validation of the expression of AND-C4 target in GBM were reinforced by immunohistochemical studies on human specimens, which revealed that it was highly expressed in GBM tissues, but undetectable in brain and LGG. These findings are in agreement with changes in EV-3 levels observed in GBM tissue and cells and supported EV-3 pathogenic relevance and suitability as a diagnostic biomarker. Furthermore, AND-C4 immunohistochemistry demonstrated its tumor specificity, underlining its potential therapeutic relevance in the complex oncogenic context of GBM.

Among the EV-3 effects counteracted by AND-C4 in GBMCs, the stimulation of cell migration deserves a comment. It is widely recognized that a feature of GBM cells is their highly infiltrative capability away from the central tumor mass into healthy brain tissue [75, 76], and this diffuse growth of cancer cells limits the effectiveness of surgical therapy [77]. Intriguingly, recent evidence suggests that actual GBM therapies, such as radiation, can aggravate GBM invasion [78]. Our finding that AND-C4 acts as a new pharmacological inhibitor of GBMC invasion underlines its potential as a tool to both block tumor spread, and to mitigate invasion-promoting effects of other therapies.

In addition, we obtained significant evidence that EV-3 and AND-C4 exerted significant, and opposite effects on GECs and PBMCs. Thus, despite EV-3 expression in both cell types remains unknown (we did not investigate it), our results demonstrate EV-3 stimulated angiogenesis of tumor-derived endothelial cells, and inhibited PBMC migration. AND-C4 was able to antagonize both pro-tumoral effects, without evidence of cytotoxicity. These results underlie the potential of EV-3 and of AND-C4 too, to affect functional properties of not only GBM cells and GSCs, but also of key cells of the GBM microenvironment, as endothelial and immune cells.

As EV-3 is an alternative splicing product, knockdown studies cannot be used to directly test that AND-C4 targets EV-3, and not Epo. To overcome this constrain, we used 16 F, an anti-Epo Ab able to target the Epo epitope responsible for its EpoR-binding, and erythropoietic actions [55, 56] and compared its actions to those of AND-C4. We found that 16 F was unable to bind rhEV-3 in SPR assays but was effective in abrogating rhEpo-induced CFU formation, in agreement with previous studies [55, 56]. The use of 16 F was functional to demonstrate that EV-3 and AND-C4 did not affect functional properties related to EpoR. In sharp contrast to 16 F, AND-C4 was unable to affect erythroid progenitor growth, reflecting that the AB-loop, essential for Epo binding to EpoR during the crucial steps of erythrocyte differentiation studies [55, 56], is not a target of AND-C4, and reinforcing EV-3, but not Epo, as the major target of this mAb.

Previous studies on the role of Epo in brain revealed that it promoted involved in neurogenesis, improved synaptic plasticity, and exerted neuroprotective effects [79]. rhEV-3 was able to mimic Epo-induced neuroprotection, by increasing the survival of cultured brain neurons from different species after different stresses [28, 80, 81]. By using the patient-derived Gliosphera, we demonstrated that AND-C4 did not affect the viability of different CNS cells. Thus, despite manipulation of EV-3 may be potentially associated with toxicity, AND-C4 failed to exert toxic effects in our models of different brain cells (NPCs, NPSCs, and ASTs).

We confirmed in vivo the anti-tumor efficacy of AND-C4 by using two mouse models of GBM, including a subcutaneous PDX, and an orthotopic intracranial xenograft. In both GBM rodent models, AND-C4 administration resulted in significant tumor volume reduction, with no observable toxicity, even when administered repeatedly. Importantly, after AND-C4 administration to mice either by intravenous or intranasal delivery, we found that it exhibited excellent tolerability, with no effects on erythropoiesis, as well as on brain and systemic toxicity.

In the orthotopic GBM model, which recapitulates the unique microenvironmental and BBB constraints of intracranial GBM, we used intranasal administration of AND-C4. This approach offers different translational advances, such as lack of invasivity, bypass of the BBB, reduced systemic toxicity, and can be also used for antibody’s delivery, with rapid absorption and higher concentrations at the tumor site [58, 59]. Moreover this method reduces peripheral exposure (and thus systemic toxicity), and, not last, it can be readily adapted to a clinical setting [58, 59]. After intranasal administration, we found that AND-C4 penetrated into the mouse brain, with selective intra-tumoral localization, and without detectable signal in the contralateral healthy hemisphere. In addition, and of relevance, it was effective not only in reducing the brain tumor mass, but also in promoting animal survival.

Overall, the results of in vivo AND-C4 treatment underscored both the specificity, and in *vivo* safety of AND-C4, and resulted in significant tumor growth inhibition, restoring of circulating lymphocyte number (reduced following intracranial GBM induction), increased apoptosis, and least but not last, significantly increased animal survival. Overall, these results revealed AND-C4 potential as a promising therapeutic tool for clinical translation. Despite our comprehensive approach, and the novel, valuable insights into GBM malignancy provided in this study, some limitations still need to be noted. First, this study did not investigate the mechanisms of EV-3 action. We are still not aware via which receptor and signaling this Epo-V exerts its tumorigenic actions. It has been proposed that Epo-Vs induce their action via the heterodimer of EpoR [82], but final proof of evidence for this hypothesis is lacking. In agreement with its structural features and previous reports [28], we found that EV-3 was unable to bind EpoR. A very recent study showed that CRLF3 is a human EV-3 receptor, and, through this binding, it was able to exert protective effects on rotenone-induced apoptosis in human neuron-like cells [83]. As Epo has been amply demonstrated to exert neuroprotection in different species including humans [83–88], and rhEV-3 appeared to share neuroprotective actions at similar rhEpo concentrations [83], the targeting of EV-3 by AND-C4 would enable to save Epo neuroprotective activities, though inhibiting EV-3 pro-oncogenic ones. To the best of our knowledge, up to now, no specific EV-3 receptor (not shared with Epo) has been recognized. Despite the possible contribution of different Epo receptors in the pro-tumorigenic actions of EV-3 and considering that Epo did not share the oncogenic actions of EV-3 in patient-derived GBM cells, the possibility of novel, yet unidentified receptor(s) mediates the actions of EV-3 in GBM should not be ignored. Future research integrating receptor studies with signaling analyses are needed and will shed light on the mechanisms that differentiate EV-3 actions from Epo-induced ones. Second, we did not evaluated EV-3 glycosylation in the present study. This post-translational modification can result in significant alterations to the overall three-dimensional structure of a protein, thus influencing biological effects, half-life, and immunoreactivity [89]. Notably, aberrant glycosylation emerged as an alteration fundamental in neoplastic properties of different cancers [90, 91], including GBM [92]. Thus, future studies will be necessary to address this limitation. Third, our patient GBM sample may appear limited, and this may affect the generalizability of the findings. It should be reminded that despite GBM accounts for over 50% of primary malignant brain tumors in human adults, this cancer is a rare, orphan disease, with an incidence lower than 0.5 cases per 10,000 person-year [93]. Despite further studies are needed, our investigation was made not only on tumor specimens, but also of their derived, primary cells from different patients, providing an almost unique and significant enrichment in our knowledge. Fourth, while our data demonstrate significant associations of EV-3 with GBM malignancy, this study deals with some brain tumors, and focused most exclusively on GBM, thus not fully representing the diversity of brain tumors, and even less of human cancers. In vitro and in vivo evaluation of EV-3 expression, functional roles, and AND-C4 sensitivity in different human malignancies is currently undergoing study in our laboratories. If these studies will provide evidence for EV-3 pro-oncogenic role in different tumors, future perspectives of AND-C4 targeting of other orphan cancers will be promising.

## Conclusions

Collectively, our data demonstrated EV-3 as a GBM-enriched, splice variant product of *EPO* transcript, able to exert potent pro-tumoral effects, and enriched in GSCs, and particularly in their microenvironment. EV-3 emerged to drive GBM proliferation, stemness, angiogenesis, and promote immune cell migration in the GBM microenvironment. These features, together with its low levels and lack of toxicity in healthy brain cells suggest its potential as a druggable target for GBM therapy. We describe the use of AND-C4, a proprietary mAb that selectively targets EV-3 while sparing erythropoiesis and physiological Epo effects, as new therapeutic option in GBM. Through in vitro, ex vivo, and in vivo models, our findings provide compelling preclinical evidence supporting AND-C4 as a safe, selective, and effective immunotherapeutic agent against GBM. By targeting EV-3, AND-C4 offers a novel mechanism to disrupt EV-3-mediated tumorigenic pathways while sparing normal hematopoiesis and brain cells. Overall, our study provides new insights and better understanding of the interaction of GBM cells and their niches, arises a previously unforeseen potential consequence of EV-3 targeting, and paves the way for future testing and validating AND-C4 mAb in human GBM.

## Supplementary Information

Supplementary Material 1.

## Data Availability

The data that support the findings of this study are available from the corresponding author upon reasonable request.

## Abbreviations

ALT: Alanine Aminotransferase
AST: Aspartate Aminotransferase
ASTR: Astrocytes
BBB: blood–brain-barrier
BLI: Bioluminescence Imaging
CNS: central nervous system
CRLF3: Cytokine Receptor Like Factor 3
CSF2RB: β-common cytokine receptor
DNA: deoxyribonucleic acid
EGFR: Epidermal Growth Factor Receptor
ELISA: Enzyme-Linked Immunosorbent Assay
EphB4: Ephrin-type-B receptor-4
Epo: Erythropoietin
Epo-Vs: Erythropoietin splice Variants
EPOR: Erythropoietin receptor
EV-3: Erythropoietin splice Variant lacking exon 3
GBM: Glioblastoma
GBMC: Glioblastoma Cells
GSCs: Glioblastoma stem-like cells
H&E: Hematoxylin and Eosin
Hb: Hemoglobin
HCT: hematocrit
HGG: High Grade Glioma
HIF1α: Hypoxia Inducible Factor 1α
i.v.: intra-venous
IDH: Isocitrate Dehydrogenase
IgG1: Immunoglobulin G1
IHC: immunohistochemistry
KD: equilibrium dissociation constant
LGG: low-grade glioma
mAb: monoclonal antibody
MGMT: O6-methylguanine-DNA methyltransferase
MNG: meningioma
MRI: Magnetic Resonance Imaging
MRP: Mutual Recognition Procedure
NPC: neuronal progenitor cells
NPSC: neural stem cells
OS: overall survival
PDX: patient-derived xenograft
PFS: Progression free survival
PK: Pharmacokinetics
RBC: red blood cells
rhEV-3: recombinant human EV-3
SPR: Surface-Plasmon-Resonance
WHO: World Health Organization
