# Supplementary material for: Revealing erythropoietin variant EV-3 as novel driving force and immunotherapeutic target in human glioblastoma
Source: J Exp Clin Cancer Res. 2025 Dec 26;45:94. doi: 10.1186/s13046-025-03620-3 (PMC13067427; doi:10.1186/s13046-025-03620-3)
Supplement: Supplementary file 1 — Supplementary Material 1. [file 13046_2025_3620_MOESM1_ESM.docx]

**Additional files**

Supplementary Figure S1

Supplementary Figure S2

Supplementary Figure S3

Supplementary Figure S4

Supplementary Figure S5

**Fig. S1. Effect of rhEV-3 and AND-C4 (C4) on human brain cell lines.** Human brain cells were treated with rhEV-3 (100 ng/mL) or/and AND-C4 (10 μg/mL) for 96h. Representative microscopic images (scale bar 100 μm), and effects of different treatments on (A) NPC, (B) NPSC, and (C) NSC proliferation. All data are reported as mean ± SD, with each sample run in triplicate.

**Fig. S2. Effect of AND-C4 on proliferation and apoptosis of patient-derived endothelial and immune cells.** GBM-derived endothelial cells (**A, B**) and peripheral blood mononuclear cells (**C, D**) were treated with AND-C4 (10 μg/mL). Cell proliferation (**A, C**) and apoptosis (**B, D**) were evaluated after 96h. Data are mean ± SD, with each sample run in triplicate.

**Fig. S3. Effect of AND-C4 on blood cells and related parameters in mice with PDX tumors. (A)** Red blood cell number, related parameters and platelet number, and **(B)** white blood cell number at day 11, and day 18 after i.v. administration of AND-C4 at different doses. Data are the mean ± S.D. RBC: red blood cells; HGB: hemoglobin; HCT: hematocrit; MCV: mean corpuscular volume; MCH: mean corpuscular hemoglobin; MCHC: mean corpuscular hemoglobin concentration; RDW: red cell distribution width; RET: reticulocyte count; PLT: platelets; WBC: white blood cells; Neut: neutrophils; Lymp: lymphocytes; Mono: monocytes; Eos: eosinophils; Baso: basophils. The dotted lines represent the reference range.

**Fig. S4. Effect of AND-C4 on liver- and kidney-related parameters in mice with PDX tumors.** Levels of circulating markers of liver function (upper panels), including albumin (ALB), aspartate aminotransferase (AST) and alanine aminotransferase (ALT), and of kidney function (lower panels), including urea and creatinine (CREA) in mice carrying PDX tumors, and i.v. treated with different doses of AND-C4 for 11 and 18 days. Data are the mean ± S.D. The dotted lines represent the physiological reference range.

**Fig. S5. Effect of AND-C4 on red blood cells and related parameters in mice with orthotopic tumors.** Red blood cell number and related parameters in mice with orthotopic GBM after intranasal administration of AND-C4. Data are the mean ± S.D. RBC: red blood cells; HCT: hematocrit; Hb: hemoglobin; MCV: mean corpuscular volume; MCH: mean corpuscular hemoglobin; MCHC: mean corpuscular hemoglobin concentration.
